# Novel candidate loci for morpho-agronomic and seed quality traits detected by targeted genotyping-by-sequencing in common bean

**DOI:** 10.3389/fpls.2022.1014282

**Published:** 2022-11-10

**Authors:** Samson Ugwuanyi, Obi Sergius Udengwu, Rod J. Snowdon, Christian Obermeier

**Affiliations:** ^1^ Department of Plant Breeding, Justus Liebig University, Giessen, Germany; ^2^ Department of Plant Science and Biotechnology, University of Nigeria, Nsukka, Nigeria

**Keywords:** *Phaseolus vulgaris*, GWAS, marker-trait association, single nucleotide polymorphisms, targeted genotyping-by-sequencing, pod shattering, seed quality, phenology

## Abstract

*Phaseolus vulgaris* L., known as common bean, is one of the most important grain legumes cultivated around the world for its immature pods and dry seeds, which are rich in protein and micronutrients. Common bean offers a cheap food and protein sources to ameliorate food shortage and malnutrition around the world. However, the genetic basis of most important traits in common bean remains unknown. This study aimed at identifying QTL and candidate gene models underlying twenty-six agronomically important traits in common bean. For this, we assembled and phenotyped a diversity panel of 200 P*. vulgaris* genotypes in the greenhouse, comprising determinate bushy, determinate climbing and indeterminate climbing beans. The panel included dry beans and snap beans from different breeding programmes, elite lines and landraces from around the world with a major focus on accessions of African, European and South American origin. The panel was genotyped using a cost-conscious targeted genotyping-by-sequencing (GBS) platform to take advantage of highly polymorphic SNPs detected in previous studies and in diverse germplasm. The detected single nucleotide polymorphisms (SNPs) were applied in marker-trait analysis and revealed sixty-two quantitative trait loci (QTL) significantly associated with sixteen traits. Gene model identification *via* a similarity-based approach implicated major candidate gene models underlying the QTL associated with ten traits including, flowering, yield, seed quality, pod and seed characteristics. Our study revealed six QTL for pod shattering including three new QTL potentially useful for breeding. However, the panel was evaluated in a single greenhouse environment and the findings should be corroborated by evaluations across different field environments. Some of the detected QTL and a number of candidate gene models only elucidate the understanding of the genetic nature of these traits and provide the basis for further studies. Finally, the study showed the possibility of using a limited number of SNPs in performing marker-trait association in common bean by applying a highly scalable targeted GBS approach. This targeted GBS approach is a cost-efficient strategy for assessment of the genetic basis of complex traits and can enable geneticists and breeders to identify novel loci and targets for marker-assisted breeding more efficiently.

## Introduction

*Phaseolus vulgaris* L. (common bean) is one of the most important grain legumes, consumed as dietary staple worldwide, especially in Latin America and Africa (Uebersax et al., 2022). Common bean has its origin in Latin America. Although the first domestication centers were in Central and Southern America, it is now widely cultivated in various regions of the world including the tropics, subtropics and temperate regions (Burle et al., 2010; Kouam et al., 2017). It is the primarily cultivated species in the genus *Phaseolus* with a broad commercial importance (Caicedo et al., 1999; Kouam et al., 2017). The importance of common bean is mainly because the seeds can serve as a substantial meal on its own and can also be used as basic ingredients in various food products. The leaves are also consumed in some parts of the world (e.g., Hillocks et al., 2006). The dry seeds and immature green pods are rich in protein, iron, essential vitamins and minerals, soluble fiber, starch and phytochemicals (Svetleva et al., 2006). These characteristics make common bean an important crop for feeding people and their livestock worldwide (Kouam et al., 2017).

Genetic diversity in common bean is categorized into two gene pools: the Andean and Mesoamerican gene pools, characterized by large seeds and small seeds, respectively (Raatz et al., 2019). Common bean genotypes are also grouped into four categories according to growth habit including determinate bushy, indeterminate bushy, determinate climbing and indeterminate climbing beans, with bush type beans being the most prevalent beans under cultivation (Assefa et al., 2019; Raatz et al., 2019). These categories are based on the type of phaseolin protein, morphological characteristics and DNA markers (Raatz et al., 2019). Common bean is very sensitive to environmental factors, with slight biotic or abiotic stress like heat- and/or drought stresses during development causing severe damage to the crop and, consequently, great loss in yield (Gross and Kigel, 1994; Santos et al., 2009; Assefa et al., 2019). Several studies have reported a broad genetic diversity among common bean genotypes (Buah et al., 2017; Gyang et al., 2017; Ahmad, 2018; Campa et al., 2018; Lioi et al., 2019; Raatz et al., 2019). This observed broad genetic base in *P. vulgaris* is indispensable in its improvement and the crop presently requires serious attention in the area of improving yield and nutritional contents, decreasing the anti-nutritional factors, improving the cooking time and resistance to biotic and abiotic stress factors (Perseguini et al., 2011; Adesoye and Ojobo, 2012).

Although most improved crop genotypes have been released through conventional breeding methods, classical breeding is often laborious and time-consuming, taking many years to release a cultivar. With advancements in DNA marker technologies, breeders have the necessary tools to speed up cultivar development *via* transfer of target genes into an important genotype through marker-assisted selection (MAS) (Das et al., 2017; Assefa et al., 2019). MAS is an efficient tool in molecular breeding, allowing the introgression of important traits into new cultivars and has been facilitated through several marker-trait association studies that have identified significant associations between markers and traits (Raatz et al., 2019). Marker-trait association studies have become a tool routinely used by researchers and breeders in the recent years for determining genomic regions affecting developmental traits, agronomic traits, resistances to pests and diseases, responses to abiotic stress, seed quality traits, and even more complex quantitative traits (Liu and Yan, 2019). Recently, a number of genome wide association studies (GWAS) have been conducted, many using the BARCBean6K BeadChip designed by Song et al. (2015), some using SSR markers and some using genotyping-by-sequencing technologies, on traits in common bean such as resistance to Bean Common Mosaic Necrotic Virus (BCMNV) (Bello et al., 2014), anthracnose (Zuiderveen et al., 2016), fusarium root rot (Hagerty et al., 2015) and Angular Leaf Spot (ALS) (Keller et al., 2015); production traits (Kamfwa et al., 2015); agronomic traits such as flowering time (Kamfwa et al., 2015; Moghaddam et al., 2016; Nascimento et al., 2018; Oladzad et al., 2019; Raggi et al., 2019), maturity time (Kamfwa et al., 2015; Moghaddam et al., 2016), growth habit, lodging and canopy height (Moghaddam et al., 2016), heat stress (Oladzad et al., 2019), pod shattering (Hagerty et al., 2015; Rau et al., 2019; Parker et al., 2020; Di Vittori et al., 2021; Parker et al., 2021a; Parker et al., 2022), and seed micronutrient content (Katuuramu et al., 2018; Diaz et al., 2020; Delfini et al., 2021; Gunjaca et al., 2021). Although these studies have helped to understand the genetic basis of some important traits in common bean, for some of these traits, the causal genes underlying these traits have largely remained unknown. Furthermore, some previous quantitative trait loci (QTL) reports for some traits in common bean have been characterized by inconsistencies across studies and a lack of comparability owing to the different marker technologies applied. This has made the identification of putative candidate gene models rather difficult, especially for phenological, yield and seed quality traits. Therefore, this calls for more marker-trait analysis using anchor markers connected to earlier studies that will facilitate the identification of genomic variants underlying important traits in common bean. Thus, this study focused on the identification of QTL underlying twenty-six important traits in common bean. The aim was to elucidate the molecular basis of these traits by applying selected targeted genotyping-by-sequencing markers, mainly derived from the commonly used BARCBean6K_3 BeadChip array containing 5398 SNP probes. The required minimum number of SNPs in a panel of Brazilian common bean cultivars for good genome coverage and satisfactory GWAS data has been estimated to be 995 (Panhoca de Almeida et al., 2020). The strategy applied here to enable cost-conscious generation of a minimum number of markers required for successful GWAS in common bean, was to derive markers from a BeadChip array which have been proven in the past to be polymorphic in a number of diverse common bean panels (Bello et al., 2014; Hagerty et al., 2015; Kamfwa et al., 2015; Moghaddam et al., 2016; Parker et al., 2021a) and to convert them into about 1000 targeted genotyping-by-sequencing markers and apply them for genotyping using a commercial genotyping service (SeqSNP, LGC Genomics; Biosearch Technologies, 2022; Gazendam et al., 2022). GBS uses restriction enzymes to reduce genome complexity and genotype multiple DNA samples, whereas targeted GBS uses probes and locus-specific PCR primers to reduce genome complexity. SeqSNP is designed for cost-effective genotyping of 100 to up to a few thousand SNP markers within an all-inclusive service including DNA extraction from leave samples and SNP calling from raw sequences. The specific objectives of our study were: a) to assemble and perform an initial phenotyping of a global diversity panel of common bean genotypes in the greenhouse for twenty-six morpho-agronomic and seed quality traits, b) to genotype the panel with 1028 SNPs using a targeted genotyping-by-sequencing (SeqSNP) technology, c) to perform a marker-trait association analysis, and d) to conduct a post-GWAS analysis for candidate gene model identification.

## Materials and methods

### Plant materials

A diversity panel of two hundred common bean genotypes including dry beans and snap beans was assembled for this study. The panel comprised genotypes from different breeding programmes, elite lines and landraces from around the world. Diversity panels are often compiled by local regions and continents (e.g., Logozzo et al., 2007; Kamfwa et al., 2015; Moghaddam et al., 2016; Campa et al., 2018; Delfini et al., 2021). African genotypes are underrepresented within these panels. Therefore, we compiled a panel, the African-European diversity panel (AED), with mainly African and European genotypes complemented by South American genotypes. Genotypes originated from several geographical regions, including Africa (n=86), Europe (n=52), South America (n=50) and Central and North America (n=8). For four genotypes, the exact origins are unknown. About 104 genotypes were selected from a panel of common bean germplasm studied by Raatz et al. (2019), including breeding lines and released varieties important in Africa and South America, and were collected from the International Centre for Tropical Agriculture (CIAT) in Uganda. Seventy-three genotypes were varieties from different African, European, North American and South American countries held at the Leibniz Institute of Plant Genetics and Crop Plant Research (IPK) gene bank in Gatersleben Germany. Thirteen genotypes were landraces collected from local markets and farmers from different states in the Northern part of Nigeria. The remaining 10 genotypes were accessions used in snap bean breeding programmes targeting the European and US market at van Waveren Saaten GmbH in Germany (see **Supplementary Table S1** for genotype list and sources).

### Phenotypic evaluation

The evaluation was conducted in a greenhouse facility at Justus Liebig University Giessen, Germany during the winter season of 2019. The experiment was laid out in a completely randomized design with two replicates per genotype, each one grown in a different pot. For each replicate two subsamples were taken (**Supplementary Table S2**). The experiment was carried out between December 2019 and April 2020 under controlled growth conditions; temperature ranged between 16°C to 20°C and a 16 hr photoperiod was applied during the growing period. There was also a turbulator in the growth chamber which ensured adequate air circulation. The seeds were planted in an 18 cm x 18 cm 5-litre plastic pots, filled with growing medium composed of a special plant growth substrate (Fruhstorfer Erde, type N; HAWITA Gruppe GmbH, Vechta, Germany), and was supplemented with inorganic fertilizer (WUXAL Universaldünger; Hauert HBG Dünger AG, Grossaffoltern, Switzerland) from the fourth week of sowing on and subsequently re-fertilized at a weekly interval until the eleventh week. The pots were spaced 20 cm apart within and between rows, and two seeds were planted per pot. The plants were properly irrigated, and the pH was maintained at about 6.0 throughout the growing period. Climbers were supported with bamboo sticks and were run down the sticks when they grew above the sticks. The bushy types were also anchored onto sticks for support to avoid lodging. Each plant was anchored separately on a stick to ensure genetic integrity.

The AED panel was harvested when the pods reached physiological maturity beginning from the eleventh week. The pods were harvested, placed in paper bags and dried to a constant weight in the oven for about 3-5 days at 40°C. The following data were collected for morpho-agronomic and seed quality traits. Growth habit, pod shattering, seed size and pod colour were recorded as categorical variables. Growth habit (GH) was scored on a scale of I-IV during the flowering stage and later confirmed at senescence, where I = determinate bushy type; II = indeterminate bushy type; III = determinate climber; IV = indeterminate climber (Assefa et al., 2019; Raatz et al., 2019). Pod shattering (PS) was scored on a scale of I-III where I = Indehiscent; II = Semi-dehiscent (intermediate); III = Dehiscent. Seed size (SS) was graded on a scale of I-III, where I = Small; II = Medium; III = Large. Pod colour (PC) was scored based on the deviation of the pod colour from green and on a scale of I-X, where I = green, II = sheen green, III = green with red patches, IV = mottled green, V = mottled purple, VI = reddish green, VII = brown, VIII = purple, IX = magenta and X = dark red. The following phenological parameters were evaluated: days to germination (GD), days to bud initiation (DTBI), days to first flowering (FFT), days to 50% flowering (FT), days to maturity (DTM) and days from flowering to maturity (DFTM). Yield related traits were also evaluated and included pod number per plant (PN), seed number per pod (ASY), total dry weight of seeded pods per plant (TDWT) (g), seed yield per plant (SN), average weight of a seeded pod per plant (APWT) (g), total dry weight of pods per plant (PDWT) (g), pod harvest index (PHI) and total seed dry weight per plant (SDWT) (g). Evaluated seed traits were 100 seed weight (HSWT) (g), seed diameter (SDia) (mm), seed length (SL) (mm) and seed dimension (SD) (mm^2^).

### Seed quality traits

The AED panel was analyzed for seed, carbon and sulfur contents. Seed sample preparation was performed by drying the seeds, pooling approximately six seeds from two plant replicates and milling into fine powder using an electric milling machine (IKA A11 basic analytical mill, IKA-Werke GmbH & Co. KG, Staufen, Germany) at maximum speed for about 1 min to obtain a homogenous powder. Analysis was carried out using the Elementar Analyzer Vario EL Cube (Elementar Americas Inc., Ronkonkoma, NY, USA) according to the company’s user’s manual. The analysis was carried out in triplicates. For each sample, seed protein content was estimated by multiplying seed nitrogen content with a conversion factor of 6.25 (AOAC, 1990).

### Phenotypic data analysis

The data collected on phenotypic traits were analyzed using R statistical software (version 1.2.5033). Summary statistics and analysis of variance (ANOVA) using fixed-factor model were conducted for every trait studied. Boxplots were created using the boxplot function in R to show phenotypic distributions based on the two major gene pools and based on major intra-gene pool subpopulations. Narrow-sense heritability is defined as the total variation in the population that is captured by additive effects. We calculated these using the heritability package in R, which estimates narrow-sense heritability based on a kinship matrix (Kruijer et al., 2015). The kinship matrix was calculated using the kinship function from the synbreed package in R (Wimmer et al., 2012). The repeatability of some of the measured traits was estimated using the repeatability function in the heritability R package. A correlation matrix was estimated to infer various associations among the phenotypic traits. Genetic analysis was performed using mean values.

### Genotyping and SNP filtering

The AED panel was genotyped using the targeted genotyping by sequencing technology (SeqSNP) by Biosearch Technologies (LGC Genomics, Teddington, UK). Leaf samples for DNA isolation were collected from the genotypes at the seventh week. Fresh leaves from the third to fourth youngest were sampled using three 96-well plate LGC plant sample collection kit and supplied to Biosearch Technologies. The panel was genotyped with 1028 selected SNP markers from which 946 markers passed quality criteria of the commercial service provider. Average effective target SNP coverage was 229x produced by 75 bp single-end read sequencing on a Illumina NextSeq 500 machine. Data preprocessing and data analysis was performed by bowtie2 alignment of reads against the common bean reference G19833v2.1, an inbred landrace line of *P. vulgaris* (G19833) derived from the Andean pool (race Peru) (Schmutz et al., 2014), variant discovery was performed using Freebayes v1.0.2-16 with a minimum coverage of 8 per locus. A vcf and excel file with variants and read numbers was received from Biosearch Technologies. The SNP dataset was filtered and SNPs retained based on i) SNPs being biallelic ii) below 50% missing marker data for accessions iii) less than 10% missing genotype data iv) less than 10% heterozygosity and v) minor allele frequency (MAF) of at least 5%.

### Population structure

Different methods were used to determine the population structure. In the first method, population structure analysis was performed using STRUCTURE 2.3.4 (Pritchard et al., 2000) with a burn-in period of 10,000 iterations and 10,000 Markov Chain Monte Carlo (MCMC) iterations using the admixture model, and an inferred clusters of K = 2 to K = 5. ΔK index was estimated to determine the most probable number of subpopulations. Principal Component Analysis and a neighbor-joining dendrogram were constructed using GAPIT in R in the second and third methods.

### Linkage disequilibrium and marker-trait association

Pairwise linkage disequilibrium among SNPs was estimated as *r^2^* and was performed alongside the association analysis using GenABEL (Aulchenko et al., 2007) and GAPIT, an R function implemented by Lipka et al. (2012). Only SNPs with < 10% missing data and > 5% MAF were used for GWAS. The association analysis was based on fixed and random model Circulating Probability Unification (FarmCPU) developed by Liu et al. (2016). This model addresses the problem of false positive control and confounding between testing markers and cofactors. In FarmCPU, the associated markers detected from the iterations are fitted as the cofactors to control false positives for testing the rest markers in a fixed effect model, and to avoid the over model fitting problem in stepwise regression, a random effect model is used to select the associated markers using maximum likelihood method (Liu et al., 2016). GWAS was also performed with a compressed mixed linear model (Zhang et al., 2010) implemented in the GAPIT R package (Lipka et al., 2012). The total principal component analysis (PCA) was set at 2 to account for population structure. SNP effects were corrected for population relatedness using false discovery rate (FDR)-corrected p-value, thereby reducing the risk of spurious marker-trait associations. Marker-trait association was considered significant above the false discovery rate (FDR)-corrected threshold of –log10 (p) > 4. However, for seed quality traits, we considered a less stringent threshold, using an arbitrary p-value –log10 (p) > 2.5 to call significant SNPs. We did this to reduce the type II error rate (false negatives) for these traits with low heritability (Kaler and Purcell, 2019). A physical map was constructed using MapChart 2.32 (Voorrips, 2002).

### QTL and candidate gene model identification

For the significant SNP markers, possible candidate gene models were identified based on proximity using a maximum of ± 100 kb distance (Raggi et al., 2019). Pairwise linkage disequilibrium (LD) for markers was constructed using HaploView 4.2 (Barret, et al., 2005) to infer haplotype blocks from SNP data. In order to determine if significant SNPs and candidate gene models were located on the same recombination blocks, LD analyses were carried out. To implicate candidate gene models affecting a trait, the list of gene models within the confidence interval of the genomic region of the SNP associated with the trait were extracted from the gff file of the common bean genome G19833v2.1 (Schmutz et al., 2014) on Phytozome v13. The possible roles of the gene models in the control of the traits were inferred based on the functional annotations for the gene models on Phytozome v13, the GO terms or best hit using blastn analysis with *Arabidopsis thaliana* gene models in the TAIR database (https://www.arabidopsis.org/ ) and published literature. A gene model was considered a candidate if the functional annotation or GO terms were related to the trait of interest.

## Results

### Phenotypic diversity and correlations between traits

**Figure 1:**
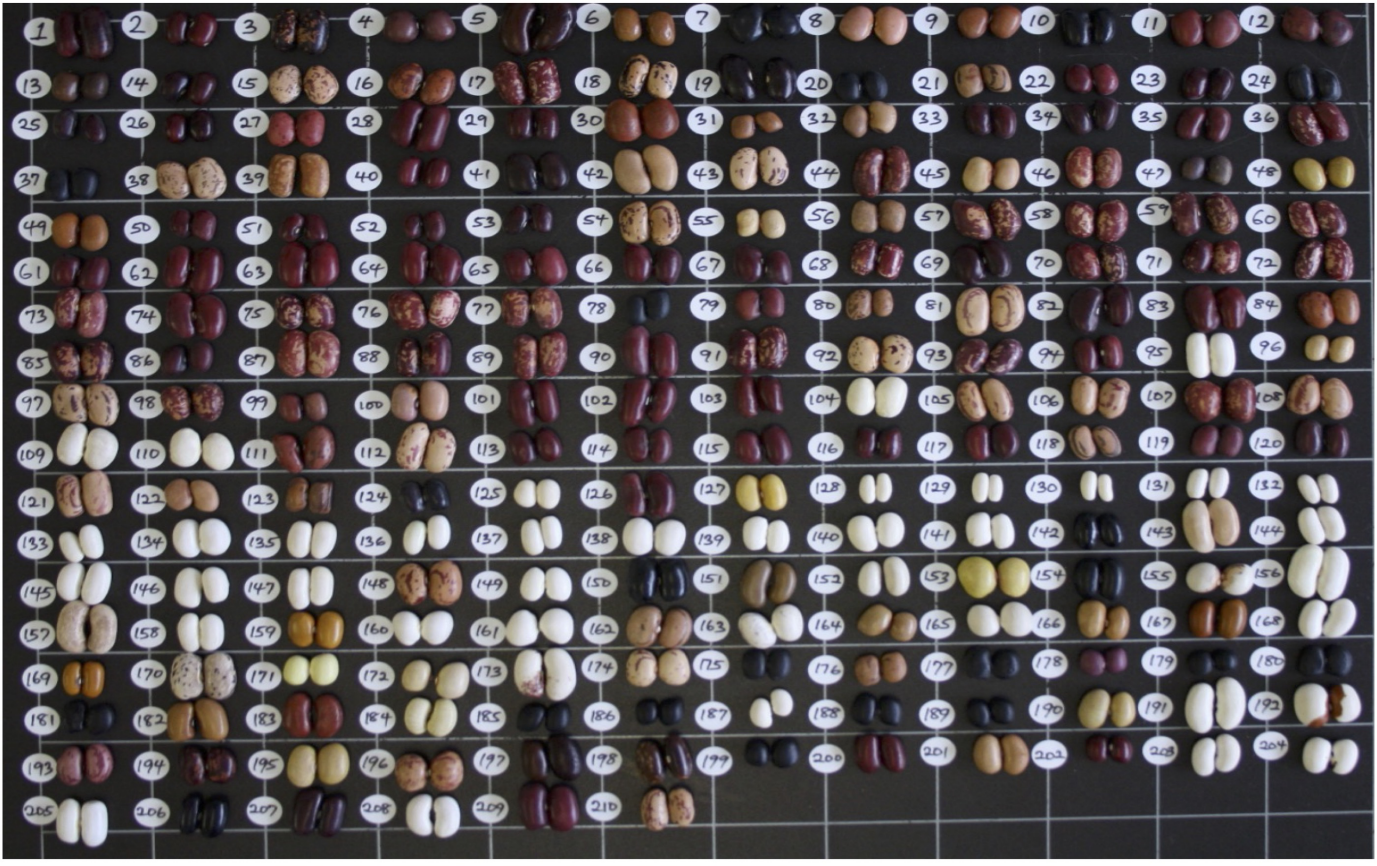
Phenotypic diversity of common bean seeds observed within the panel. Numbers correspond to genotype names in **Supplementary Table S1**.

The phenotypic expression of morpho-agronomic and seed quality traits varied widely across the AED panel (**Supplementary Table S3**). Growth habit types observed in the diversity panel included determinate bushy, determinate climbing and indeterminate climbing types. Only indeterminate bushy type was missing in the panel. The seed sizes ranged from small to large seeds. They showed highly diverse seed coat patterns and colours (**Figure 1**). Three forms of pod shattering such as indehiscent, semi-dehiscent and dehiscent forms were observed. Similarly, there were wide variations in other production, phenological and seed quality traits. Most traits measured as quantitative variables showed normal distributions (**Supplementary Figures 1**, **2**). Across the two major gene pools, the Andean genotypes had higher mean values for seed traits while the Mesoamerican genotypes had higher mean values for yield related traits (**Supplementary Figures 3**, **4**). A similar high intra-group variation was observed within both gene pools. However, different levels of variations were detected within the five intra-gene pool subpopulations (**Supplementary Table 5**, **6**). The means, coefficient of variation (CV), heritability values and ranges for the twenty-six traits measured are summarized in **Table 1**. Trait repeatability showed a broad range from 0.36 to 0.97. The marker-based estimates of heritability (narrow-sense heritability, *h^2^_r_*), ranging from 0.44 to 0.99, correlated strongly with the repeatability. Of the sixteen flowering, maturity, yield related and seed traits, 62.4% (10 traits) displayed narrow-sense heritability above 0.36, and six traits showed narrow-sense heritability over 0.7 providing the genetic basis for the GWAS. The three flowering time traits displayed the highest narrow-sense heritability (> 0.7), while eight yield related traits showed relatively low narrow-sense heritability compared with other groups (**Table 1**). The analysis of variance revealed highly significant differences (P < 0.001) for the quantitatively measured traits (**Supplementary Table S2**). However, because the heritability calculations are based on one greenhouse experiment only, they might be overestimations. Thus, the following QTL analysis results should be treated as preliminary and with caution unless highly significant or previously reported.

**Table 1:** Summary of phenotypic traits measured for 192 common bean genotypes.

Trait (unit)	Abbreviation	Mean ± SD	CV (%)	Min.	Max.	Heritability (*h^2^_r_*)	Repeatability
Growth habit (4 categories)	GH	–	–	1	4	–	–
Pod colour (10 categories)	PC	–	–	1	10	–	–
Pod shattering (3 categories)	PS	–	–	1	3	–	–
Seed length (mm)	SL	12.84 ± 2.4	18.6916	8.11	18.89	0.900	0.910
Seed diameter (mm)	SDia	7.85 ± 1.12	14.2675	4.15	10.35	0.890	0.890
Seed size (3 categories)	SS	–	–	1	3	–	–
Seed dimension (mm^2^)^1^	SD	102.37 ± 29.01	28.3384	37.86	183.22	–	–
100 seed weight (g)	HSWT	39.83 ± 17.42	43.7359	8.42	110.94	0.590	0.750
Seed number (seeds)	SN	41.35 ± 30.07	72.7207	2.00	130.50	0.561	0.744
Seed dry weight (g)	SDWT	12.69 ± 5.74	45.2325	1.22	35.37	0.356	0.458
Average seed yield (seeds)	ASY	3.56 ± 1.12	31.4607	1.50	6.95	0.600	0.670
Pod number (pods)	PN	10.85 ± 5.95	54.8387	1.00	37.00	0.581	0.653
Average pod weight (g)	APWT	1.8 ± 0.62	34.4444	0.33	4.05	0.591	0.629
Pod dry weight (g)	PDWT	4.64 ± 1.94	41.8103	0.81	13.35	0.335	0.357
Total dry weight (g)	TDWT	17.33 ± 7.33	42.2966	2.03	48.72	0.320	0.425
Pod harvest index	PHI	0.73 ± 0.06	8.2192	0.50	0.83	–	–
Germination time (days)	GD	9.2 ± 1.27	13.8043	8.00	16.50	0.447	0.436
Days to bud initiation (days)	DTBI	48.79 ± 21.37	43.8000	34.00	140.00	0.997	0.971
First flowering time (days)	FFT	52.61 ± 0.65	1.2355	37.00	140.00	0.994	0.961
Flowering time (days)	FT	54.02 ± 19.87	36.7827	44.00	140.00	0.995	0.965
Days to maturity (days)	DTM	110.94 ± 22.16	19.9748	84.50	180.00	0.955	0.965
Days from flowering to maturity (days)	DFTM	56.86 ± 8.23	14.4741	42.00	84.50	0.673	0.697
Seed sulfur content (%)	Sulfur	2.4836 ± 0.0654	0.0003	2.4836	2.8620	–	–
Seed protein content (%)^2^	Protein	23.70 ± 4.48	18.9030	9.70	36.60	–	–
Seed carbon content (%)	Carbon	42.58 ± 1.02	2.3955	39.59	44.69	–	–
Seed carbon to nitrogen ratio	C:N	11.65 ± 2.39	20.5150	7.27	27.49	–	–

SD is the standard deviation of the mean; CV is coefficient of variation; Min. and Max. are the maximum and minimum range for a trait, *h^2^_r_* is the marker-based estimation of narrow-sense heritability from individual plant replicates according to Kruijer et al. (2015); ^1^Calculated by multiplying seed diameter and seed length. ^2^calculated from seed nitrogen (N) content (%) by using a conversion factor of 6.25.

**Figure 2:**
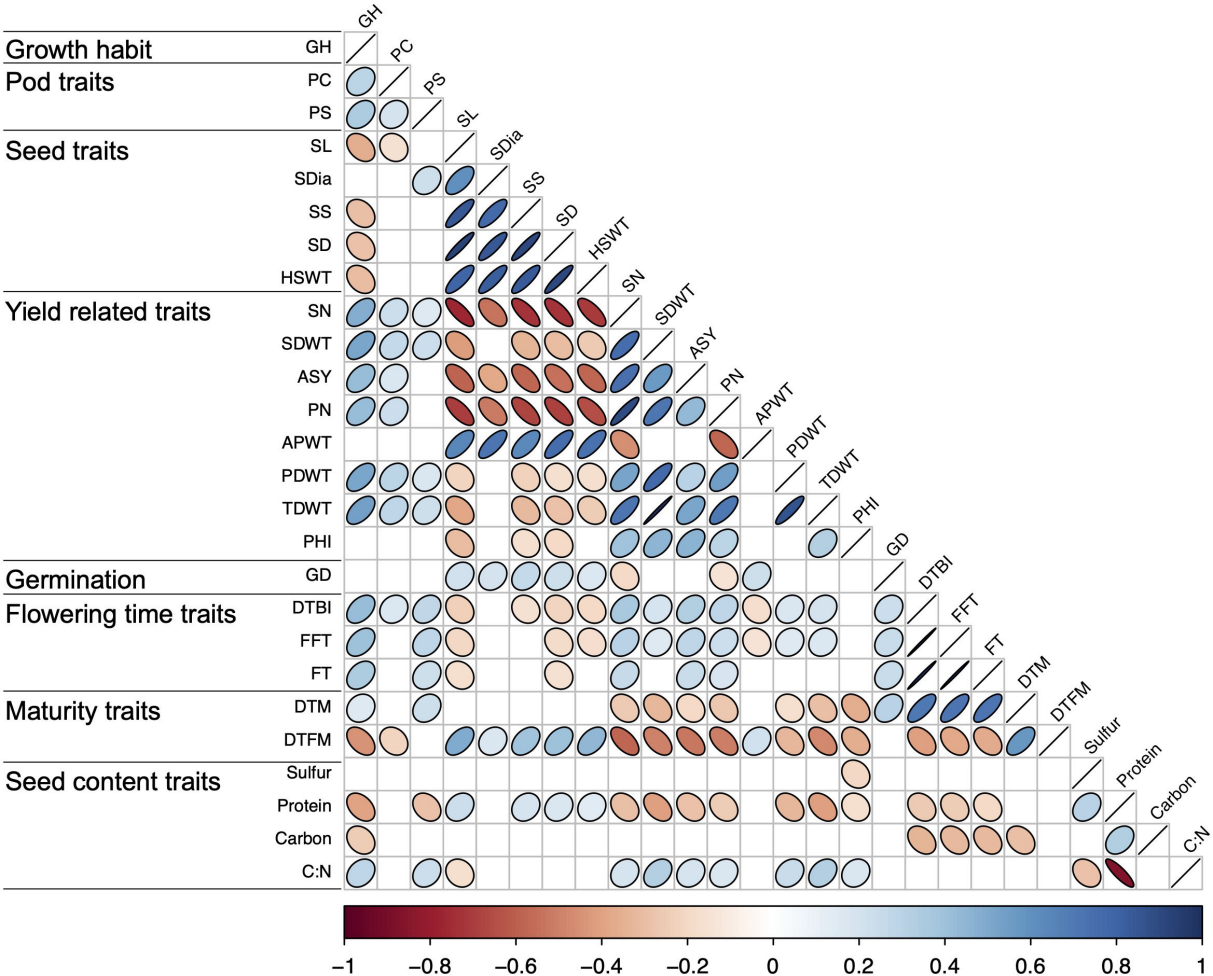
Correlation matrix for all phenotypic traits above r = 0.19. See **Table 1** for trait abbreviations.

**Figure 3:**
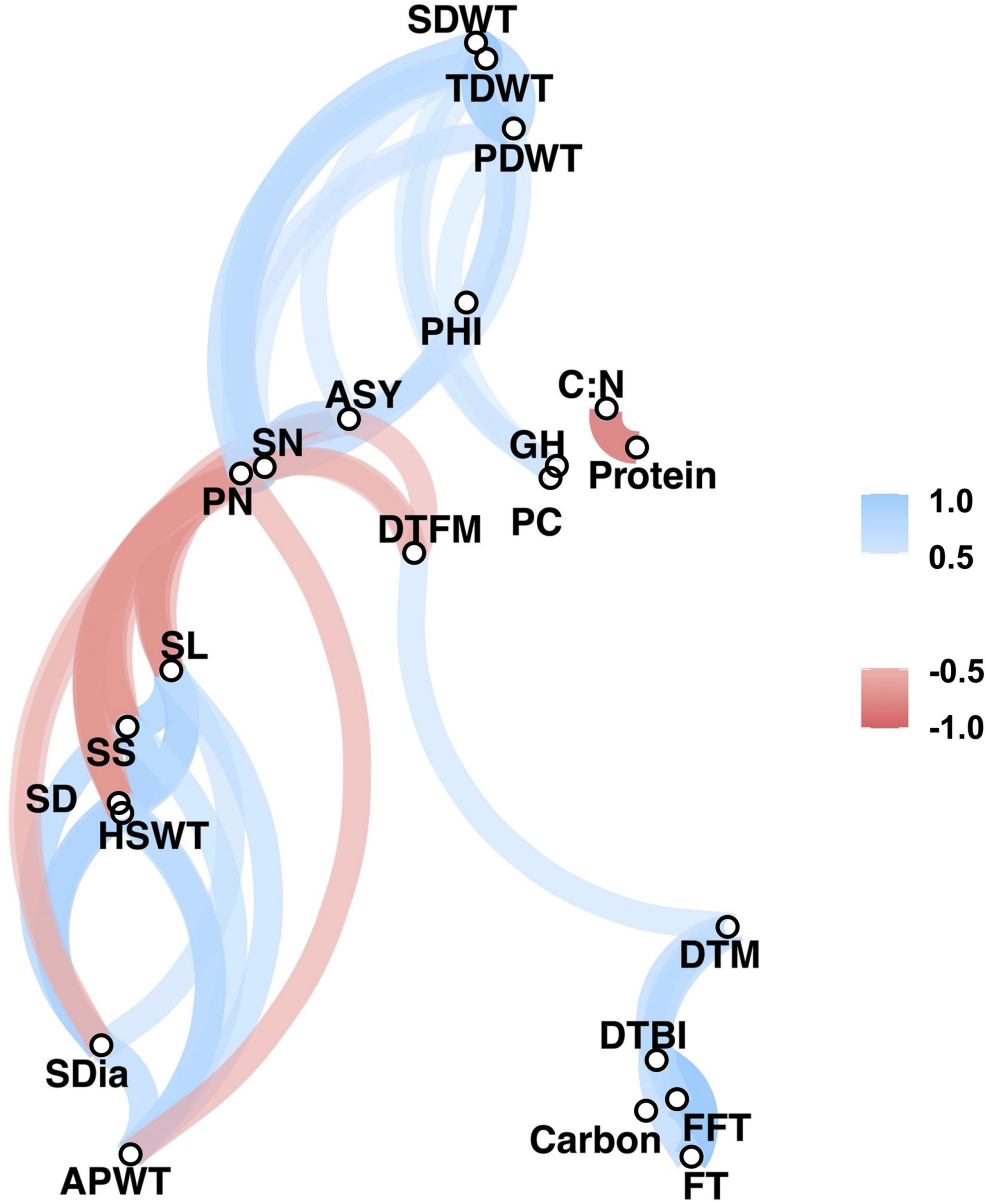
Correlation network plot for all phenotypic traits above r= 0.5. See **Table 1** for trait abbreviations.

Significant positive and negative correlations were observed between a number of traits (**Figures 2**, **3**). Correlation coefficients ranged from -0.77 to 0.98. Positive correlations were found between growth habit (GH) and pod shattering (PS), flowering time (DTBI, FFT, DTM, FT, DFTM) and yield related traits (PN, SN, TDWT, SDWT, PDWT, ASY). For abbreviations of the traits, see **Table 1**. A negative correlation was observed between growth habit and seed protein content. Flowering time traits correlated positively with pod shattering (PS) and yield related traits. Also, days to maturity correlated positively with yield related traits.

### Genotyping

A total of 2500 SNP flanking sequences were selected from previous studies (Kamfwa et al., 2015; Nascimento et al., 2018; Oladzad et al., 2019; Raatz et al., 2019), some of which were reported to be associated with important traits and were part of the SNPs on the BARCBean6K BeadChip designed by Song et al. (2015). The flanking sequences were aligned to the common bean reference genome G19833v2.1 (Schmutz et al., 2014) using ncbi-blast 2.12.0+; filtered and processed in order to determine the positions and alleles of the SNPs in the reference genome. After this step, about 2234 SNPs were selected initially for SeqSNP primer design for targeted genotyping-by-sequencing by Biosearch Technologies. From the initial design, about 4468 primer pairs were developed to target the 2234 SNPs. Subsequently the primer pairs were re-filtered based on criteria such as high specificity, distance between SNPs (bp), previous report of trait associations and polymorphisms in the genomic regions. A final set of primer pairs targeting 1028 SNPs were developed for genotyping the AED panel. Overall, 92% of the targets passed the quality criteria for final analysis at LGC Genomics. Four percent of the genotypes in the diversity panel had over 50% missing marker data and were removed. In these genotypes, which were all Nigerian common bean landraces (NCB4, NCB7, NCB8, NCB9, NCB11, NCB12, NCB15, and NCB16), over 50% of the SNPs failed, suggesting that they may represent a different *Phaseolus* species and/or are genetically very different to the *P. vulgaris* genotypes from which the SNP markers were originally derived. These landraces appeared morphologically different from the rest of the genotypes and thus were excluded from the analysis. The remaining 192 genotypes, including the other five Nigerian landraces, passed the filtering criteria. The SNP dataset was filtered based on SNPs being biallelic and having less than 10% missing genotype data, less than 10% heterozygosity and at least 5% minor allele frequency (MAF). After SNP quality control, a total of 867 polymorphic SNPs were retained and used in the subsequent genetic analysis. The smallest number of polymorphic SNP markers were recorded on chromosome 4 with 48 polymorphic SNPs, while chromosome 9 had the highest number of polymorphic SNPs (101). The SNP distribution across the common bean chromosomes after filtering is shown in **Supplementary Table S4**.

### Population structure

**Figure 4:**
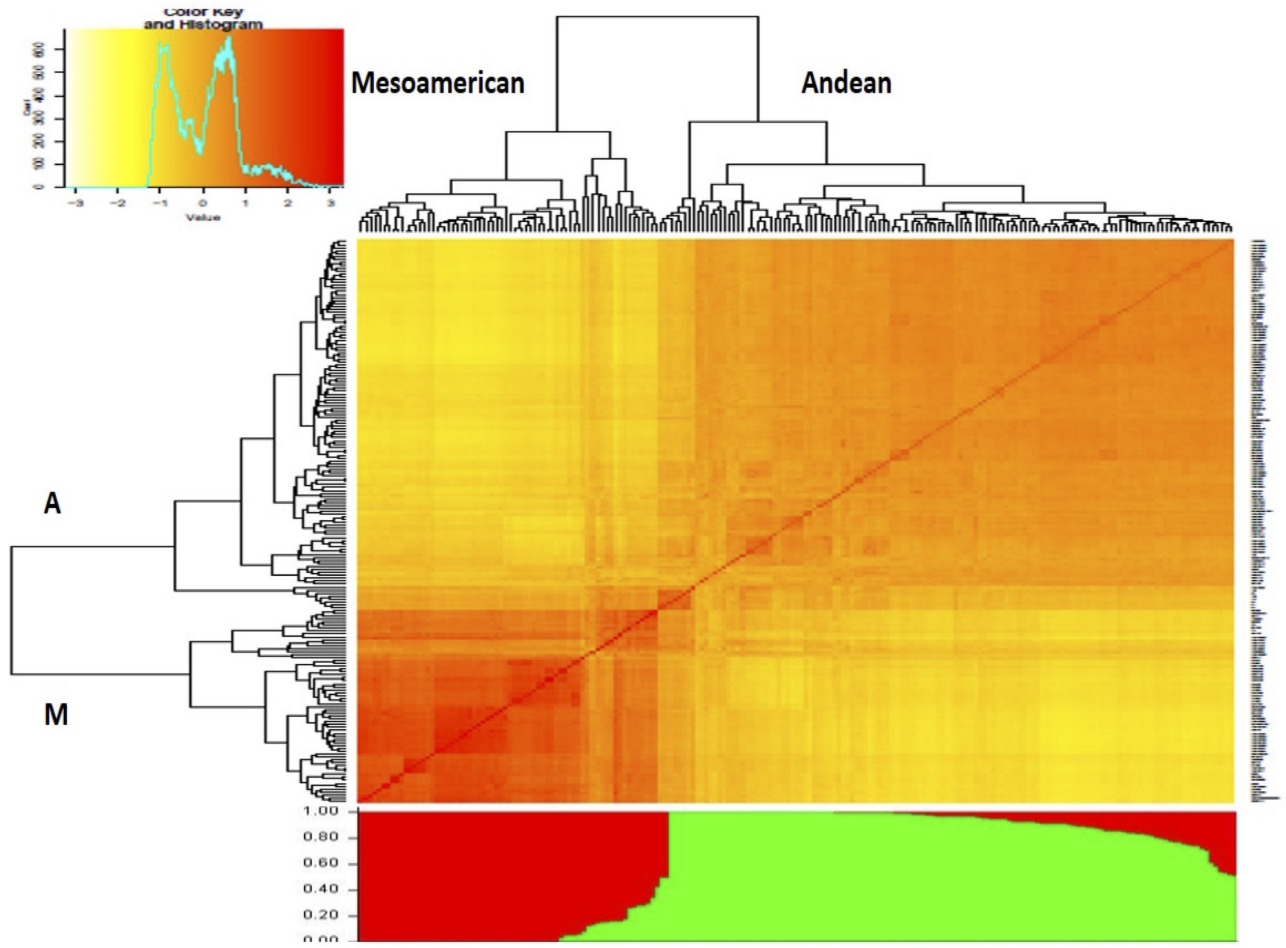
Dendrogram clustering 192 Phaseolus vulgaris accessions based on the genetic distances (above) and structure analysis bar plot (below). Both analyses divide the panel into two subgroups, designated “A” and “M”, representing the Andean and Mesoamerican clades, respectively.

The number of subgroups within the diversity panel was evaluated using Structure and GAPIT. In the Structure, the Q-matrix was defined by a ΔK index with a peak at K = 2, indicating the number of main subgroups which represent the Andean and Mesoamerican gene pools. The heatmap from GAPIT showed the two major clusters while the bar plot diagram showed each individual in k-coloured segments with lengths equivalent to each of the subgroup (**Figure 4**). The population structure was also confirmed by the principal component analysis and NJ dendrogram (**Supplementary Figures 7**, **8**) generated from GAPIT. The PCA scatter plots indicated the two distinct subgroups belonging to Andean and Mesoamerican gene pools. The distribution of the genotypes within the two major clades assigned 126 genotypes to the Andean gene pool and 66 genotypes to the Mesoamerican gene pool.

### Linkage disequilibrium

The linkage disequilibrium analysis was conducted with the 867 polymorphic SNPs for the 192 genotypes and all chromosomes using the squared-allele frequency correlation (*r^2^*). LD decay in the population was estimated from the mean *r^2^* as a function of inter-SNP distance. Calculations indicated that LD values decayed with the genetic distance in the population, with an average *r^2^* of about 0.4 for very close markers < 500 Kb, and decays to about 0.1 for markers as distant as 5 Mb. The average *r^2^* drops to 0.25 above 2 Mb; an average *r^2^* of about 0.2 extends up to 4 Mb. **Supplementary Figure 9** shows the average and chromosome-based LD decay in our panel.

### Marker-trait associations for agronomically important traits

**Figure 5:**
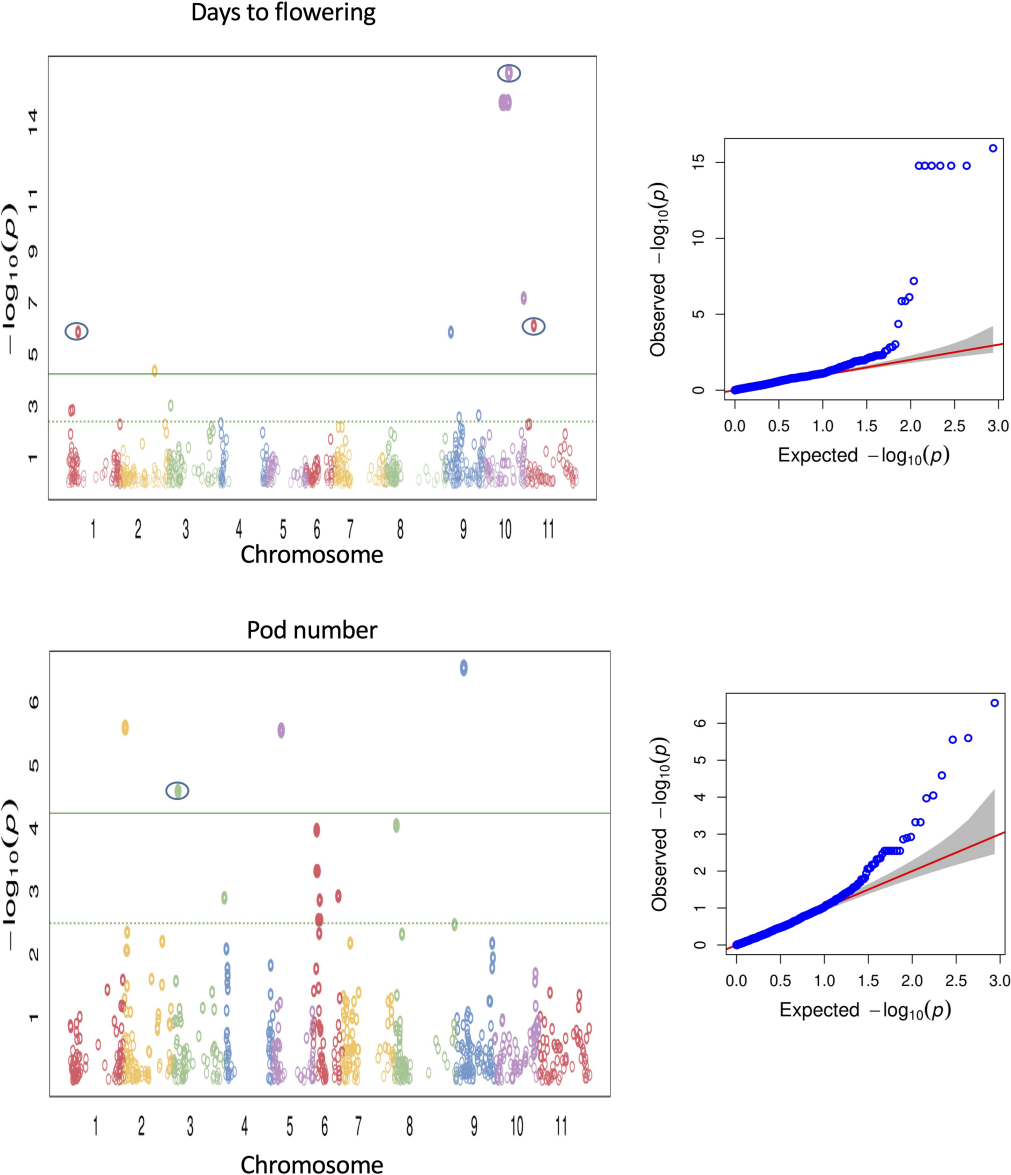
Manhattan and QQ plots for GWAS using FarmCPU for flowering time and pod number. The green line is the FDR cutoff value to call a significant peak. Peaks enclosed in circles indicate SNPs associated with QTL overlapping with previously reported QTL.

Association of the phenotypic traits with 867 polymorphic SNPs was implemented using the fixed and random model Circulating Probability Unification (FarmCPU). Also, association analysis was performed with a compressed mixed linear model (Zhang et al., 2010) implemented in the GAPIT R package (Lipka et al., 2012). We report here only QTL detected by FarmCPU as the MLM model in GAPIT detected a subset of major QTL previously reported for the investigated traits, whereas the QTL detected by FarmCPU largely agreed with previously reported QTL. It has been reported before that the MLM model can lead to false negatives by overcompensating for population structure and kinship (e.g., Sun et al., 2016). FarmCPU has been developed to remove the confounding effects between population structure, kinship, and quantitative trait nucleotides and prevents model over-fitting, and controls false positives simultaneously (Liu et al., 2016). Thus, it seemed to be most adequate with our common bean panel harbouring a strong population structure. Multiple genomic regions were identified to be associated with 16 morpho-agronomic and seed traits (see **Supplementary Table S5**). About 67 SNPs across different chromosomes showed associations with 14 morpho-agronomic traits above the FDR threshold. The lowest P-value (1.16 x 10^-16^), indicating the strongest association between a marker and a trait, was observed on chromosome Pv10 for flowering time, explaining about 38.5% variation for the trait (**Figure 5**). Low P-values were also observed for many other traits, for example pod number (see **Figure 5**). For seed quality traits, we identified 7 SNPs associated with seed protein and sulfur contents. For some traits there were no significant associations above the FDR threshold, such as days from flowering to maturity, total dry weight, pod dry weight, seed dry weight, seed weight, 100 seed dry weight, seed size and seed dimension. The Manhattan plots for the different SNP-trait associations are shown in **Supplementary Figures 10**–**12**. **Supplementary Table S5** summarizes the significant SNP-trait associations observed from the marker-trait association analysis. Although all SNP-trait associations should be treated as preliminary as they are based on one greenhouse environment, we identified a number of previously reported QTL regions for 12 out of 26 traits (peaks enclosed in circles in **Figure 5** and **Supplementary Figures 10**-**12**).

### Candidate gene models underlying morpho-agronomic and seed quality traits

**Figure 6:**
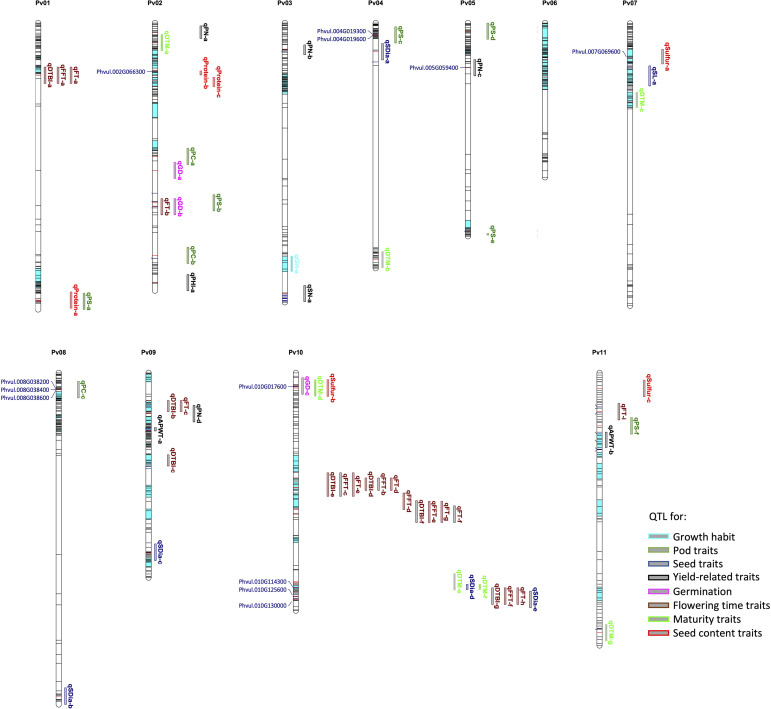
Physical map showing SNP distribution, significant markers, QTL and candidate genes identified for sixteen traits across the common bean genome. The loci in dark and red colour indicate all the SNPs used in the genotyping; the red lines indicate significant markers while the bar segments in light blue indicate haplotype blocks containing more than one SNP. The QTL and candidate genes are aligned on the right and left of the chromosomes, respectively. Refer to **Supplementary Table S6** for details on QTL and candidate genes.

Below we describe ten selected traits and QTL regions where we could identify novel candidate gene models potentially relevant for better understanding of the biological mechanisms underlying the studied traits including flowering time, yield related, seed quality and pod traits (see **Figure 6**). In total, we identified 12 novel candidate gene models underlying ten traits in common bean (**Table 2**). Gene Ontology (GO) terms associated with candidate gene models within the intervals of QTL associated with phenological traits include flower development and late flowering for flowering time traits, and nutrient mobilization and seed germination for germination time. Terms such as seed development, seed size and maturation were associated with the candidate gene models for seed traits. Plant organ morphogenesis, flower, embryo and seed development and biomolecule synthesis were associated with yield related trait like seed number, while amino acid, nitrogen and sulfur metabolism were related to gene models associated with seed quality. However, some of the candidate gene models are poorly described in literature. For details on all candidate gene models see **Supplementary Table S6**.

**Table 2:** QTL for which novel candidate genes were identified for morpho-agronomic and seed quality traits in a panel of 192 diverse common bean accessions.

Trait^1^	QTL ID	Selected major candidate gene model ID(s)	QTL report^2^	Candidate gene models from literature	Tissue-specificity^3^	Reference(s)	Comments
PS	qPS-c	Phvul.004G019300; Phvul.004G019600	Yes/No	–	Flower, pod, seed	Rau et al. (2019)	Suggested a minor QTL on Pv04 that is assumed to modulate pod dehiscence
PC	qPC-c	Phvul.008G038200; Phvul.008G038400; Phvul.008G038600	Yes/No	Phvul.008G262700	Flower, pod, seed	Garcia-Fernandez et al. (2021)	QTL identified for pod colour in this region
FT/FFT/DTBI	qFT-h/qFFT-f/qDTBI-g	Phvul.010G125600	Yes/Yes	Phvul.010G142900	Flower	Nascimento et al. (2018)	A QTL identified for days to flowering in previous studies overlaps for a similar trait in our study
GD	qGD-c	Phvul.010G017600	Yes/Yes	Phvul.010G017600	Plant embryo, seed, fruit	Moghaddam et al. (2016)	The authors identified this gene model as a candidate for seed weight in common bean
SDia	qSDia-e	Phvul.010G130000	No	–	Flower, seed	Wang et al. (2015)	Functions in regulating seed size
PN	qPN-c	Phvul.005G059400	Yes/No	–	Flower, pod, seed	Kamfwa et al. (2015)	
Protein	qProtein-b	Phvul.002G066300	Yes/No	–	Flower, seed	Gunjaca et al. (2021)	A QTL for nitrogen content was identified on this chromosome
Sulfur	qSulfur-a	Phvul.007G069600	No	–	Flower, seed	Depuydt and Vandepoele (2021)	Orthologs reported to modulate sulfur metabolism in Arabidopsis

^1^Refer to **Table 1** for trait abbreviations, ^2^QTL reported previously on the same chromosome, but exact position unknown/QTL reported previously on the same chromosome and co-localizing, ^3^Information for the tissues where genes are expressed in were obtained for gene orthologs in *A. thaliana*.

For flowering time traits such as FT, FFT and DTBI, one candidate gene model was observed within the overlapping QTL confidence interval. Located within the QTL on chromosome Pv10 is Phvul.010G125600 which encodes a protein involved in PHYTOCHROME-DEPENDENT LATE-FLOWERING (Endo et al., 2013). For pod shattering, two candidate genes are located near the QTL on chromosome Pv04, Phvul.004G019300 and Phvul.004G019600. The candidate gene models are associated with cell wall structures in pods that result to shattering in *A. thaliana*, *Glycine max* and *Phaseolus vulgaris* (Gille et al., 2011; Kou et al., 2012; Dong et al., 2017; Rau et al., 2019; Di Vittori et al., 2021). For germination time, Phvul.010G017600 is a major candidate gene model which is localized within the QTL on chromosome Pv10. The functional annotation showed that the ortholog of this gene model in Arabidopsis affects seed germination by controlling the metabolic efflux and protein storage in seed, associated with the onset of suspensor and endosperm programmed cell death and early nutrient mobilization to nourish the growing embryo (Sreenivasulu and Wobus, 2013; Moghaddam et al., 2016). We mapped three gene models within the QTL associated with pod colour which encode proteins involved in the production of different plant pigments (Gonzalez et al., 2008; Garcia-Fernandez et al., 2021). All three, Phvul.008G038200, Phvul.008G038400 and Phvul.008G038600, are myb domain protein 113 encoding gene models that regulate anthocyanin biosynthesis (Gonzalez et al., 2008). Phvul.010G130000 was identified as candidate gene model for seed diameter and it is associated with seed development. In Arabidopsis, the ortholog of Phvul.010G130000 functions in the regulation of seed size *via* cell expansion (Wang et al., 2015). Phvul.002G066300 is localized within the QTL on chromosome Pv02 for seed protein content. Its Arabidopsis ortholog is associated with amino acid and organo-nitrogen metabolism (Depuydt and Vandepoele, 2021). For sulfur content a QTL was found harbouring Phvul.007G069600 encoding a cytochrome b561/ferric reductase transmembrane protein family which is reported to modulate sulfur metabolism in Arabidopsis (Depuydt and Vandepoele, 2021).

## Discussion

### Phenotypic and molecular characterization reveals wide variation within common bean

This study assembled a panel of common bean genotypes important in different breeding programmes around the world. The phenotypic evaluation of the genotypes revealed wide variations in twenty-six traits from different trait categories including growth habit, pod traits, seed traits, yield related traits, germination, flowering time, maturity and seed content. A wide diversity in seed size was observed which can be attributed to the presence of the two major gene pools: Andean and Mesoamerican, with large seeds belonging to the Andean and the small seeds belonging to the Mesoamerican. Wide variations in phenological and production traits were also observed and consistent with previous findings (Kamfwa et al., 2015; Moghaddam et al., 2016). The seed quality analysis revealed a wide range of estimated protein content. Previously, the protein content of common bean grown in different environments has been reported to range from 16% to 35% for landraces and for improved varieties (Miles et al., 2015; Celmeli et al., 2018; Rezende et al., 2018). Our study recorded a wider range (9% to 37%) potentially explained by the larger sample size and the wide diversity of our genotypes consisting of landraces, breeding lines and cultivars from a world-wide selection. Population genomic analyses based on SNPs grouped the genotypes into two major clusters with 66% of genotypes corresponding to the Andean gene pool and 34% corresponding to the Mesoamerican gene pool. This observation is similar to what has been reported in various genetic diversity studies involving common bean (Buah et al., 2017; Gyang et al., 2017; Ahmad, 2018; Campa et al., 2018; Lioi et al., 2019; Raatz et al., 2019). These two major clusters represent gene pools which are known to originate from two independent domestication events after partial reproductive isolation. Although the Andean gene pool is usually considered to be about three times less diverse than the Mesoamerican gene pool (Bitocchi et al., 2013; Schmutz et al., 2014; Cichy et al., 2015; Campa et al., 2018; Lioi et al., 2019), the selected genotypes showed a similar within-group genetic diversity in our study, making the selected panel a well-suited basis for genetic analysis. The broad genetic base observed in this study is essential for common bean improvement since a high genetic diversity is indispensable for successful breeding programmes. The high genetic diversity observed within the assembled common bean panel provides sufficient variations for genetic studies of agronomically relevant traits.

### Adverse correlations of pod shattering and seed content traits with yield traits require genetic dissection of underlying factors

High negative values were found between traits from different trait categories such as seed traits, yield related traits and maturity traits, e.g. between hundred seed weight (HSWT) and seed number (SN) and seed dry weight (SDWT) and days from flowering to maturity (DFTM) as has been reported before (e.g. Kamaluddin and Ahmed, 2011; Ambachew et al., 2015). Growth habit showed correlation with traits related to flowering time and yield. The bushy types flowered earlier but were less productive than the climbers. They showed a smaller biomass which bore fewer floral buds. This was unlike the climbers which were larger in size and bore numerous flowers which would eventually develop into pods. This type of correlations between flowering time traits and yield components, and between growth habit and yield components have been reported before (Kamfwa et al., 2015; Moghaddam et al., 2016; Oladzad et al., 2019). Murgia et al. (2017) reported correlations of pod shattering with 100 seed weight and pod colour, but no correlations with seed number, seed dry weight, pod dry weight and growth habit in a panel of introgression lines produced from a wild Mesoamerican genotype with extensive pod shattering into an Andean genotype exhibiting no pod shattering. Pod traits, growth habit, yield related-traits and maturity traits were negatively correlated with seed protein content, indicating that the bushy types are mostly indehiscent and had higher protein contents than the climbers. This is similar to what Murgia et al. (2017) observed and suggests that larger biomass and pod shattering occurs at a cost as resources expended towards shattering and canopy height limits the resources available for storage in plant tissues.

### Genome wide association analysis in common bean can be successfully performed using a limited number of targeted GBS markers

An average of four significant marker-trait associations were identified for a total of 16 phenotypic traits. This was achieved, although with a comparatively low number of polymorphic SNP markers (867) by applying a targeted genotyping-by-sequencing approach. In recent years, GWAS has mainly been performed in common bean using the BeanK_3 Bead array with 3900 to 4900 polymorphic SNP markers (e.g. Kamfwa et al., 2015; Jain et al., 2019) or by using GBS type approaches using 30,000 to 50,000 polymorphic SNP markers (e.g. Raggi et al., 2019; Garcia-Fernandez et al., 2021). However, common bean is an autogamous plant with very long blocks of markers in linkage disequilibrium ranging from 500 kb to 1.15 MB in populations of around 200 genotypes (e.g. Nadeem et al., 2020; Delfini et al., 2021) and a small genome size of around 600 MB (Schmutz et al., 2014). Thus, the required numbers of non-redundant markers covering the same LD blocks is expected to be in the range of 500 to 1000 markers for genotyping without losing mapping precision in GWAS. We calculated an average LD decay of up to 4 MB at *r^2^* = 0.2. The LD decay for chromosomes Pv06, Pv07, and Pv11 in the panel was exceptionally low (**Supplementary Figure 9**) suggesting that these chromosomes require a limited number of markers for successful GWAS. Diniz et al. (2019) and Bhakta et al. (2015) also found extended LD blocks in the distal region of the long arm of chromosome Pvu06. In our analysis, 61.1% of all SNP markers (530) applied in GWAS were not in LD with neighboring markers and 89.6% of all inherited blocks were represented by single marker coverage (**Figure 6**). Thus, we could successfully apply a cost-effective targeted GBS approach by selecting a low number of markers proven to be polymorphic in earlier studies and by avoiding markers which are physically closely anchored on the reference genome. SeqSNP also has the advantage that it is accessible for researcher which have no experience in GBS library preparation and have no specialized laboratory equipment available. SeqSNP is an all-inclusive service including DNA extraction from leave samples, assay design from customer supplied SNP sequences, library production, Illumina sequencing, alignment and SNP calling. Costs for 190 to 377 genotypes and up to 1000 SNP markers are from approximately 7 to 15 US $ (e.g., Zhang et al., 2020). This is less than half the costs required for a commercial standard GBS service not including DNA extraction. In an earlier study, Nascimento et al. (2018) had applied about 384 SNPs in a marker-trait association analysis and successfully identified significant SNPs associated with flowering time and subsequently mapped three candidate genes thought to be affecting flowering in common bean. These results indicate that a relatively low number of SNPs could be used in marker-trait association study in common bean if carefully selected and well-spaced. For instance, we identified significant SNP associations in genomic regions that were not densely covered with SNPs, owing to the inclusion of SNPs from genomic regions reported to show high polymorphisms in common bean (Song et al., 2015; Raatz et al., 2019).

GWAS identified QTL associated with sixteen traits, some of which are novel while some overlap with previously reported QTL. QTL were reported as overlapping if located within the confidence interval defined as the peak SNP ± 100 kb of the associated SNP in our study. For instance, the QTL qFT-c and qDTBI-b/c observed for flowering time were not reported in earlier studies, while the QTL on chromosomes Pv01, Pv02, Pv10 and Pv11 are located on the same chromosomes and/or are overlapping with QTL previously reported for flowering time traits (Blair et al., 2006; Mukeshimana et al., 2014; Kamfwa et al., 2015; Moghaddam et al., 2016; Diaz et al., 2020; Keller et al., 2022). However, QTL for flowering time have been consistently reported on chromosome Pv01 across many geographic regions. Although a significant QTL was identified on chromosome Pv01 for flowering time in our study, the QTL only explained minimal phenotypic variance in our panel. Apparently, the effect of this QTL is limited in the broader genetic background of our study, which involved the two common bean gene pools, in contrast to previous reports where either bi-parental populations or only one of the gene pools were used in the analysis. For maturity time, we identified three novel QTL on chromosomes Pv02, Pv07 and Pv10. Similarly, three out of the six QTL identified for pod shattering are new while the other three QTL on chromosome Pv02, Pv04 and Pv05 have been reported in earlier findings (Hagerty et al., 2016; Rau et al., 2019; Parker et al., 2020; Di Vittori et al., 2021). We mapped a major QTL linked to growth habit on chromosome Pv03, which co-localizes with a QTL associated with growth habit reported by Keller et al. (2022). In addition, our study identified many QTL for yield related traits although this was not expected for traits with low heritability as in our study, the panel was only phenotyped in one greenhouse environment. Some of these QTL were previously reported (Blair et al., 2006; Wright and Kelly, 2011; Mukeshimana et al., 2014; Kamfwa et al., 2015; Oladzad et al., 2019). Co-localization of significant SNPs was observed for different traits. For example, there was a co-localization of QTL for flowering time traits. Similarly, a QTL on chromosome Pv10 was associated with both days to maturity and seed sulfur content. Co-localization of QTL for different traits in common bean was previously reported for FT and DTM (Kamfwa et al., 2015) and for lodging, canopy height and growth habit (Moghaddam et al., 2016). Co-localization of QTL for different traits could be due to pleiotropy or linked genes residing in the same region (Kamfwa et al., 2015).

Cultivation of beans showing a determinate bushy growth habit with photoperiod insensitivity (day-neutral response) is beneficial for commercial production as it results in a shortened in a shortened life cycle and tolerance to mechanical harvesting (Repinski et al., 2012). The dissection of the genetic basis underlying the growth habit will allow for its manipulation in breeding programmes. In general, indeterminate plants develop vegetative buds at terminal meristems and stem apices that regulate the development of new nodes with leaves and produce inflorescence in axillary meristem. Consequently, the extension of stem length is indeterminate. On the other hand, the determinate types grow to a limited stem length and terminate with floral buds. Genes involved in different developmental pathways in the apical meristem have been a target for understanding the molecular basis of transition from vegetative to reproductive stage (Repinski et al., 2012). In the present study, we report a QTL with a major effect on chromosome Pv03 for growth habit. Previous studies by Repinski et al. (2012) and Moghaddam et al. (2016) reported a QTL for this trait on chromosome Pv01. More recently, Keller et al. (2022) reported many QTL for growth habit in a diverse panel, including QTL on chromosomes Pv01, Pv03 and Pv06. One of the QTL on chromosome Pv03 co-localizes with our QTL and our finding is in agreement with what Keller et al. (2022) observed where the Pv01, Pv03 and Pv06 QTL distinguished the determinate bushy types from the other growth types.

### Marker-trait association reveals novel candidate gene models controlling agronomically important traits in common bean

Candidate gene models are discussed below in detail especially for some agronomically important traits exhibiting high heritability such as pod shattering and flowering time related traits, as our study revealed preliminary QTL harbouring novel interesting candidate gene models for these traits.

Pod shattering is an important trait in legumes, desired for reduction in yield losses at maturity. Gioia et al. (2013) identified a candidate gene, *PvIND*, on chromosome Pv02 that is located near the *St* locus to affect pod strings, a factor influencing pod dehiscence. Another study reported a major locus on chromosome Pv02 which explained 32% of variation in pod suture string in a recombinant inbred population and mapped *St* locus to bordering PvIND on Pv02 (Hagerty et al., 2016). In contrast to these reports and based on an introgression line population, Rau et al. (2019) and Di Vittori et al. (2021) mapped one single major locus on chromosome Pv05, qPD5.1-Pv, and minor loci on other chromosomes associated with loss of dehiscence in the Andean gene pool. More recently, Parker et al. (2022) mapped a major QTL for pod strings within the vicinity of *PvIND* on chromosome Pv02 in a recombinant inbred population, suggesting that *PvIND* controls pod string in common bean. The authors reported that tandem duplication of *PvIND* and retrotranspon insertion controls stringless pods in common bean. However, pod string is one of the pod traits that influences pod shattering and it is expected that there are more loci that underlie other pod traits, which together determine pod shattering. For example, major loci on chromosome Pv03 and Pv08 are associated with significant reductions in pod shattering in the Mesoamerican and Andean gene pools, and more loci controlling other pod traits such as fibre development in pod sutures and walls have also been reported (see Parker et., 2020, Parker et al., 2021b). We mapped six QTL regions including both previously reported loci on Pv02 and Pv05 in our world-wide panel by GWAS. This suggests that in contrast to the bi-parental populations used before, which exhibited limited genetic and phenotypic diversity for pod shattering, our panel is more suitable for mapping multiple pod shattering loci relevant for breeding in common bean. Previously, Rau et al. (2019) and Di Vittori et al. (2021) reported Phvul.005G157600 as a major locus underlying pod shattering in common bean. However, this gene model is located outside the confidence interval of the QTL qPS-e on Pv05, located about 500 kb away from this QTL. In our study, two candidate genes are reported for pod shattering. These candidate genes are located within the QTL qPS-c on chromosome Pv04 and are considered strong candidates due to their roles in pod shattering in other species. For example, the Arabidopsis ortholog of Phvul.004G019600 encodes a cellulose-synthase-like C4 protein which functions in cell wall modifications resulting in silique dehiscence in Arabidopsis (Dong et al., 2017), whereas Phvul.004G019300 is involved in xyloglucan metabolic pathway (Gille et al., 2011). Pod shattering is highly associated with genes encoding cell wall modifications and hydrolases. This has been shown in crops such as *Vicia sativa* L. where pod shattering was attributed to the dissolution of cell wall in the ventral suture of the pod due to the breakdown of glycosidic bonds of pectin and cellulose by the encoded proteins (Dong et al., 2017). Additionally, shattering genes have shown involvements in cell wall modifications in other crops such as soybean (Dong et al., 2014; Funatsuki et al., 2014), cowpea (Suanum et al., 2016; Lo et al., 2018) and Medicago sp (Ferrándiz and Fourquin, 2014).

Flowering is a complex trait and thought to be controlled by a network of genes that regulate different flowering pathways in plants. Several genes with potential roles for flowering in common bean were suggested previously (Kamfwa et al., 2015; Moghaddam et al., 2016; Nascimento et al., 2018; Raggi et al., 2019; Diaz et al., 2020). However, reports of candidate genes for flowering in common bean across different studies have not been consistent. Although it is possible that different QTL/genes control flowering in common bean under different environments and genetic backgrounds, we identified the gene model Phvul.010G125600 as a novel candidate gene model for flowering time. The candidate gene model is located within the +/- 100 kb interval of the QTL on chromosome Pv10, which explained a total variance of 38.5% for flowering time in the population. The Arabidopsis ortholog is a PHYTOCHROME-DEPENDENT LATE-FLOWERING gene model associated with flowering (Endo et al., 2013). A QTL harbouring this gene model has been reported previously. Nascimento et al. (2018) identified a QTL with a weak effect which overlaps with this QTL on chromosome Pv10 for flowering time in eighty Brazilian breeding lines. They suggested the gene model Phvul.010G142900 as a potential candidate gene model from within an +/- 3.5 MB interval around the significant SNP. Phvul.010G142900 encodes an early flowering 3 protein that is associated with the initiation of flowering in Arabidopsis. However, this gene model is about 400 kbp away from our peak SNP marker and unlikely to be involved in flowering-time modulation in the AED panel.

In conclusion, the present study has successfully expanded the genetic information on agronomically important traits in common bean. The phenotypic data showed high diversity within the study panel, capturing important genotypes mainly from the African and European continent that are early maturing, high yielding with high protein contents, among other important characteristics. We provided further insight into the genetic architecture of some important traits in common bean by successfully identifying sixty-two preliminary QTL and twelve novel candidate genes potentially underlying these traits. Our study also revealed that pod shattering is controlled by multiple loci with six QTL contributing to the observed variation in our panel. Pod shattering is an important breeding target in some market classes of common bean exhibiting high levels of pod shattering. The loci involved in pod shattering resistance have been found to vary between different gene pools (Parker et al., 2021a). We identified new loci involved in pod shattering and the alleles could be transferred between gene pools based on the identified flanking SNP markers. However, the panel was evaluated in a single greenhouse environment and the findings should be substantiated in detailed field studies across different field environments. Some of the detected QTL and a number of candidate genes elucidate the understanding of the genetic nature of these traits and provide the basis for further studies. Furthermore, the study showed the possibility of using a limited number of SNPs in performing marker-trait association in common bean by applying a highly scalable targeted GBS approach. This targeted GBS approach is a cost-efficient strategy for assessment of the genetic basis of complex traits and can enable geneticists and breeders to identify novel loci and targets for marker-assisted breeding more efficiently.

## Data availability statement

The datasets presented in this study are included in the article/**Supplementary Materials**. Further inquiries can be directed to the corresponding author.

## Author contributions

SU, CO and OU conceived the idea. SU performed the experiments. SU, CO and OU developed the methodology. RS sourced the funding. SU and CO performed data curation and analyzed the data. SU and CO drafted the manuscript. SU, CO, OU and RS revised and approved the final manuscript. All authors contributed to the article and approved the submitted version.

## Funding

SU received funding from the German Academic Exchange Service (DAAD) through the programmes: DAAD In-Country/In-Region Scholarship (grant number: 57423580) and Short-Term Research Grant in Germany (grant number: 57500260).

## Acknowledgments

SU appreciates the German Academic Exchange Service (DAAD) for their funding during his study programme. We thank Annette Plank, Stavros Tzigos and Roland Kürschner for their assistance during the phenotypic evaluation. We are also grateful to Benjamin Wittkop and Stjepan Vukasovic for their help with the Elementar Analyzer. We thank Sven E. Weber and Iulian Gabur for help with R scripting. We appreciate the Department of Plant Science and Biotechnology, University of Nigeria Nsukka and Prof. Paul Bayeri of Crop Science Department, University of Nigeria Nsukka for their valuable comments and suggestions during this work. We thank Thomas Meyer-Lüpken from Van Waveren for critical review of the manuscript. We thank Van Waveren, IPK and CIAT, Uganda for providing the seeds.

## Conflict of interest

The authors declare that the research was conducted in the absence of any commercial or financial relationships that could be construed as a potential conflict of interest

## Publisher’s note

All claims expressed in this article are solely those of the authors and do not necessarily represent those of their affiliated organizations, or those of the publisher, the editors and the reviewers. Any product that may be evaluated in this article, or claim that may be made by its manufacturer, is not guaranteed or endorsed by the publisher.
