# Supplementary material for: Novel candidate loci for morpho-agronomic and seed quality traits detected by targeted genotyping-by-sequencing in common bean
Source: Front Plant Sci. 2022 Nov 10;13:1014282. doi: 10.3389/fpls.2022.1014282 (PMC9685177; doi:10.3389/fpls.2022.1014282)
Supplement: Supplementary file 1 [file DataSheet_1.pdf]

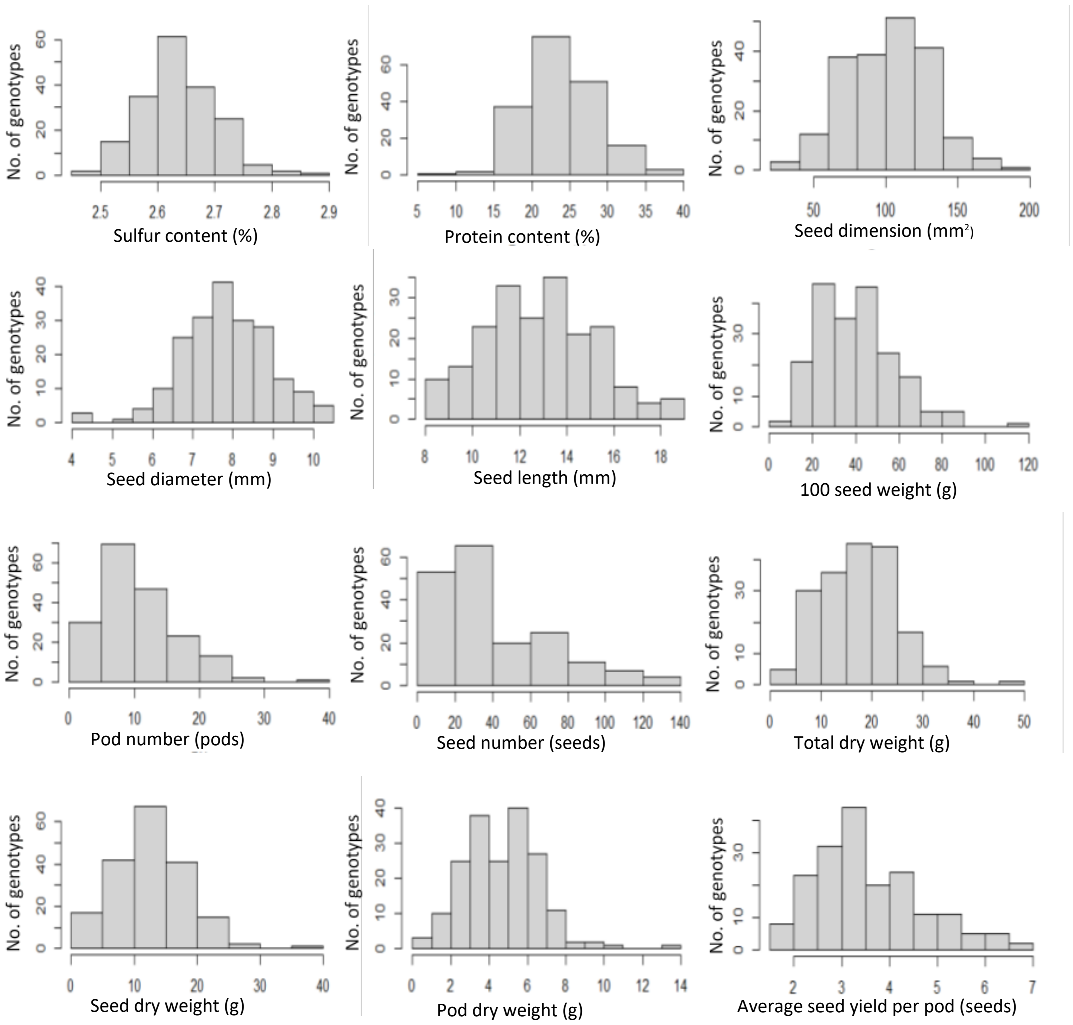

Supplementary Figure 1 Histogram showing the normal distribution of 192 genotypes for some seed quality, seed characteristics and yield traits

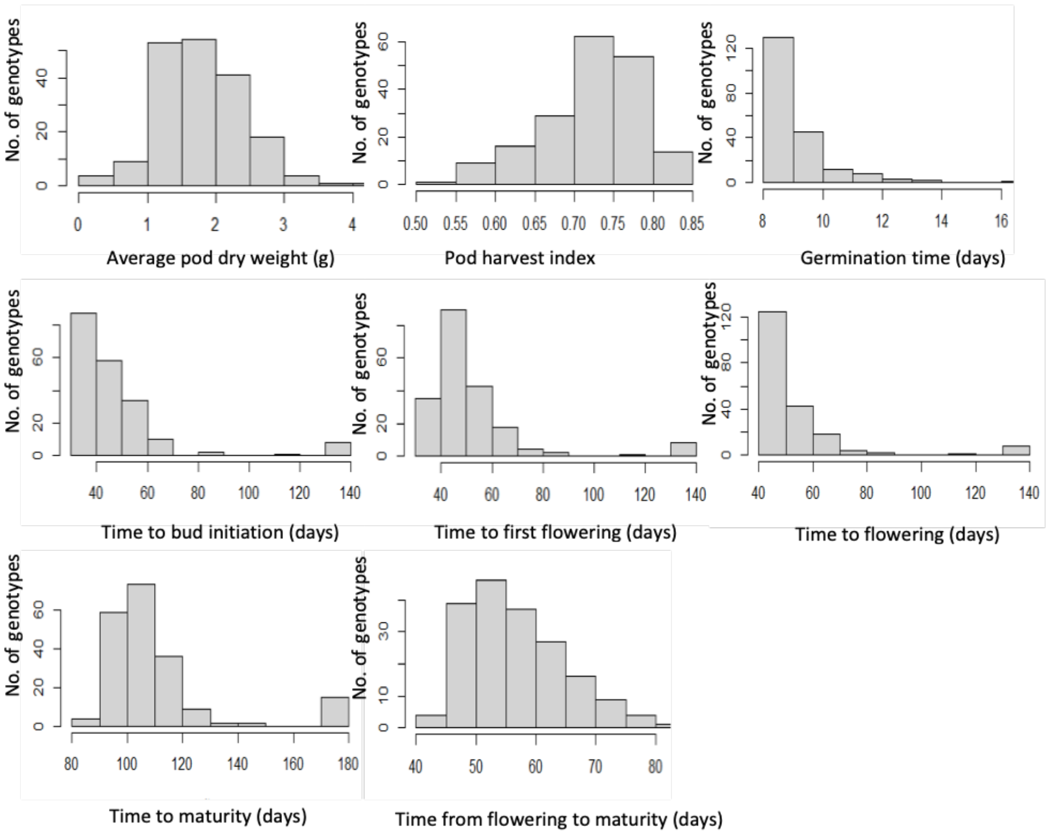

Supplementary Figure 2 Histogram showing the normal distribution of 192 genotypes for some yield and phenological traits

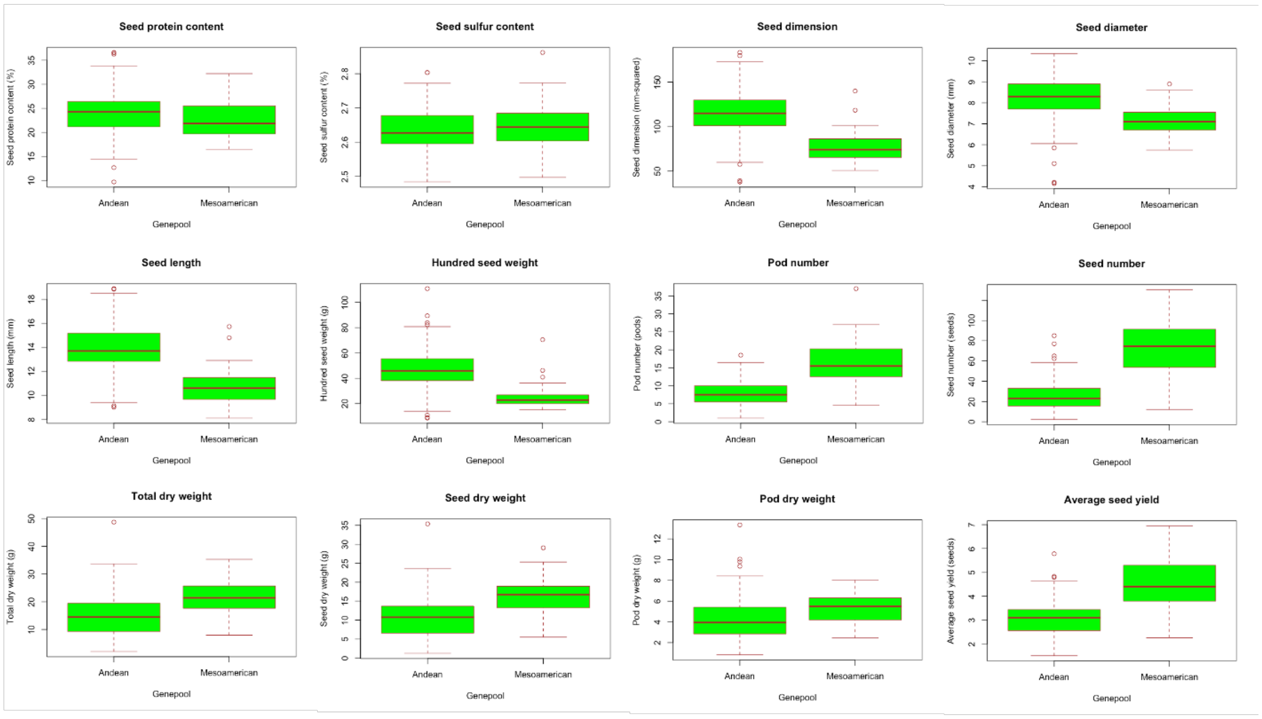

Supplementary Figure 3 Boxplots showing phenotypic distributions within the two major gene pools in common bean for some seed quality, characteristics and yield traits

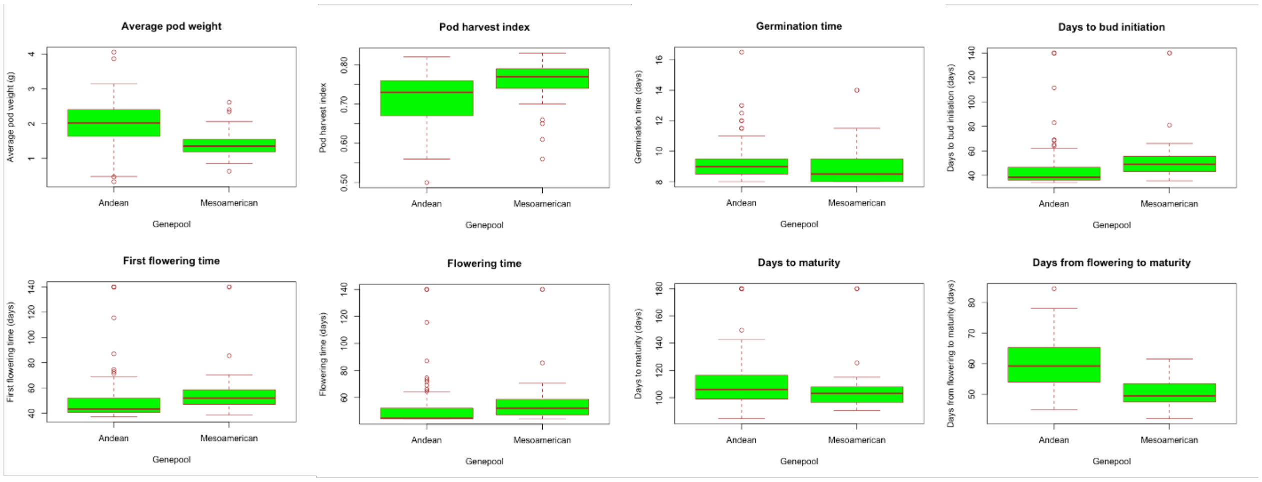

Supplementary Figure 4 Boxplots showing phenotypic distributions within the two major gene pools in common bean for some yield and phenological traits

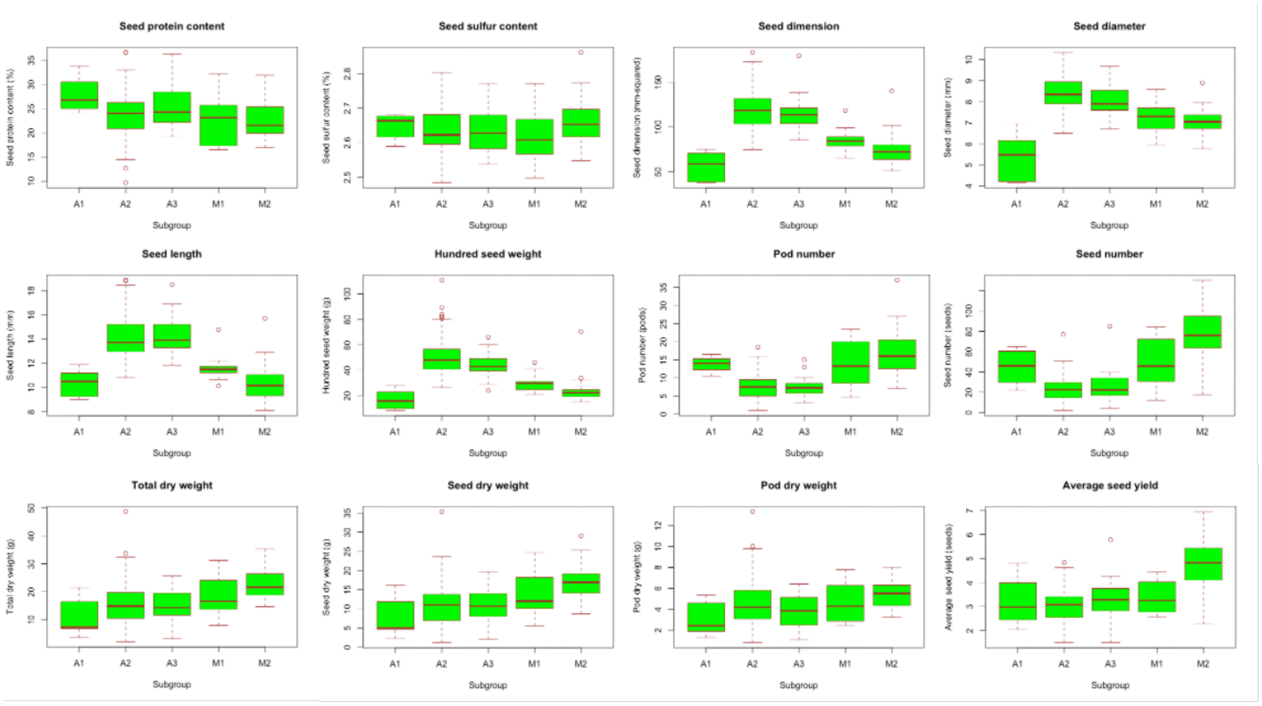

Supplementary Figure 5 Boxplots showing phenotypic distributions within major subpopulations of Andean and Mesoamerican genepools for some seed quality, characteristics and yield traits

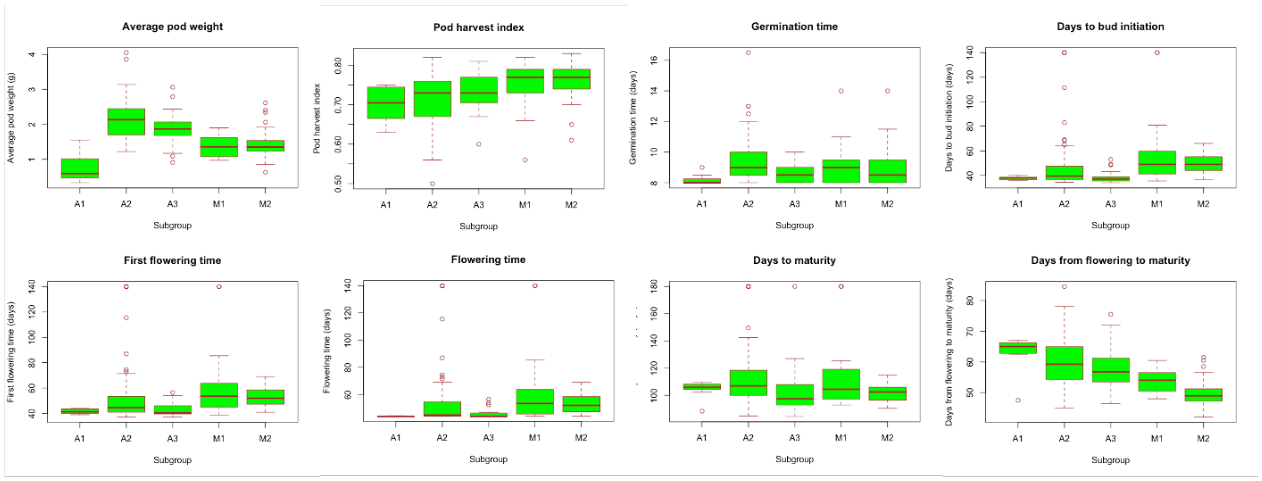

Supplementary Figure 6 Boxplots showing phenotypic distributions within major subpopulations of Andean and Mesoamerican genepools for some yield and phenological traits

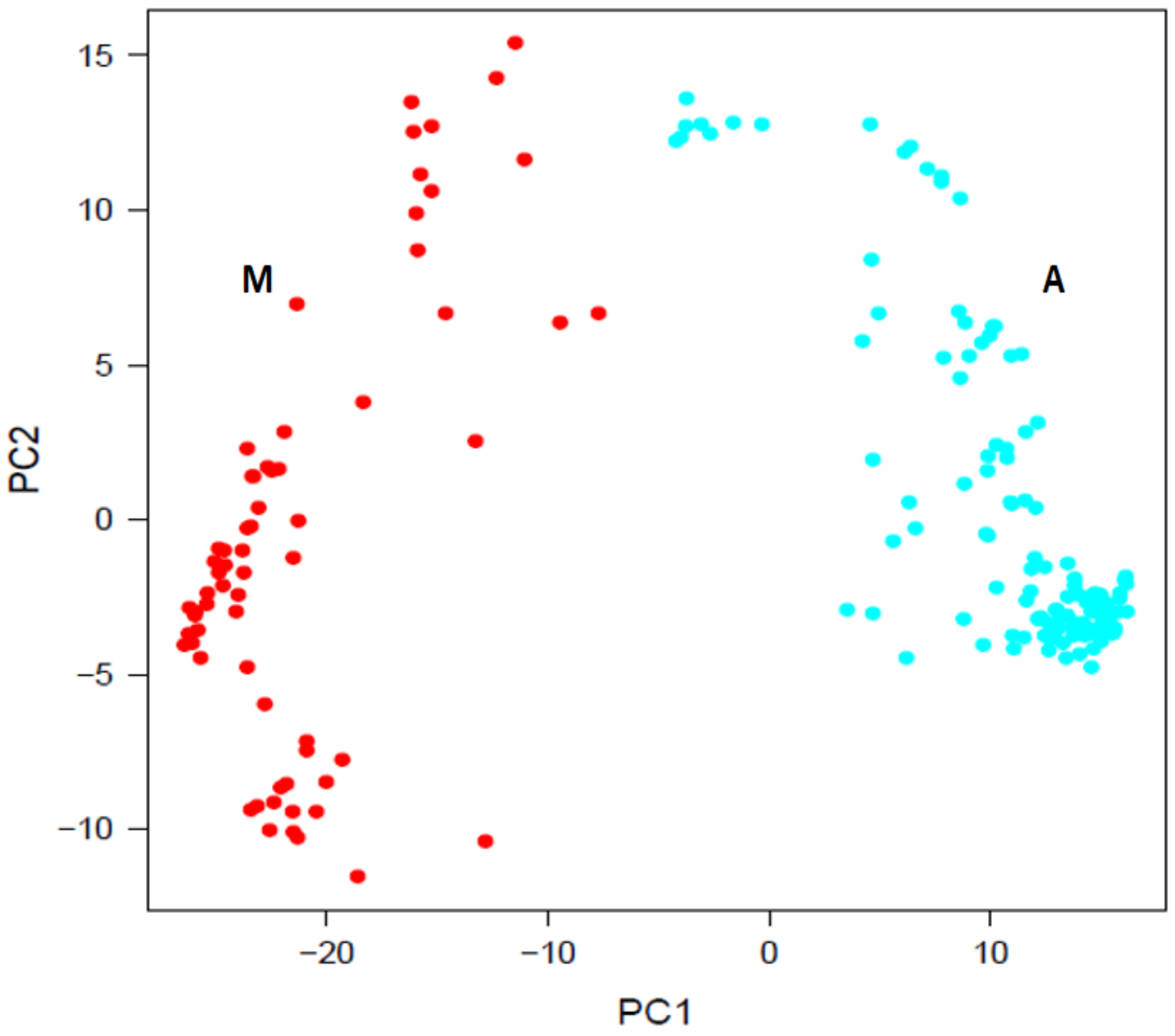

Supplementary Figure 7 Principal component graph dividing the panel into two distinct subgroups A and M representing the Andean and Mesoamerican clades

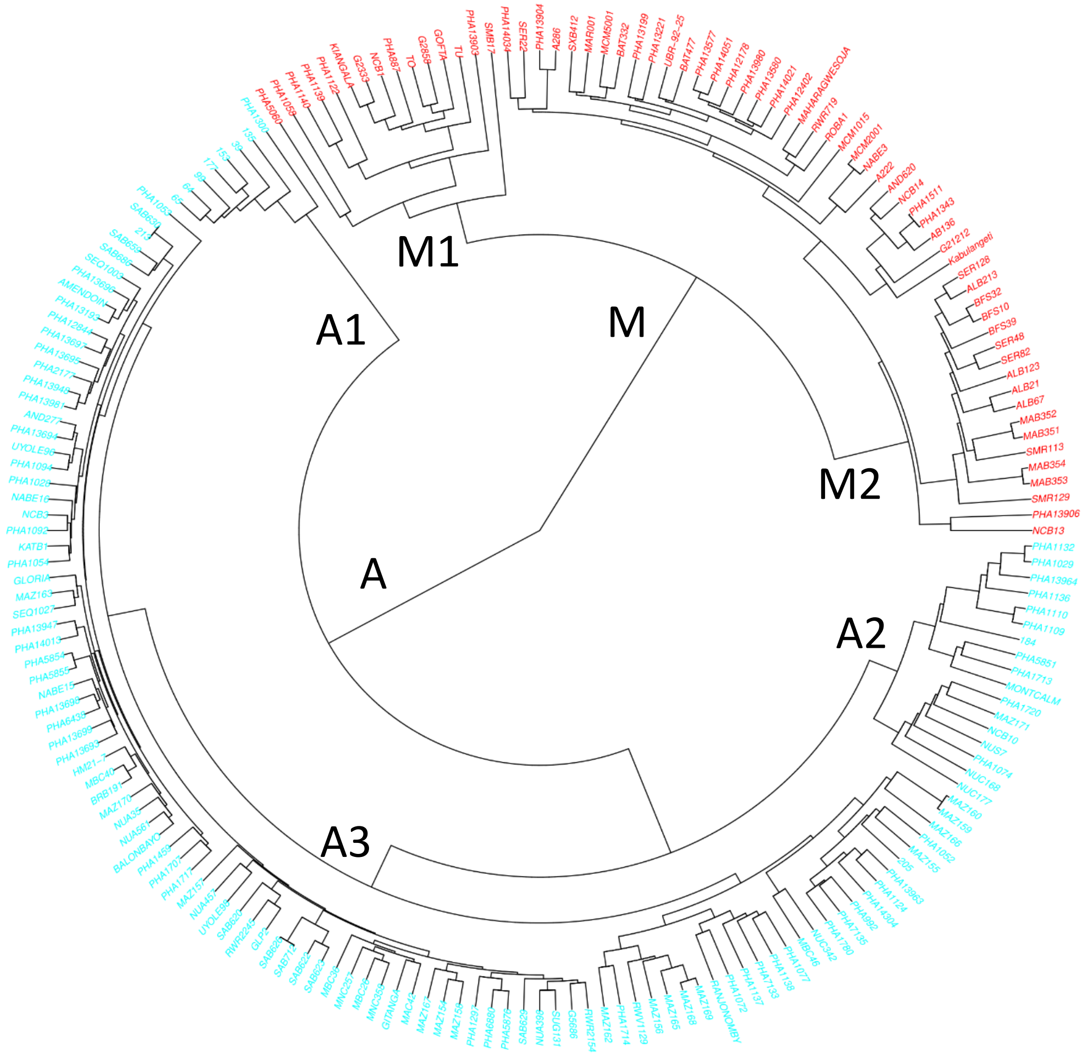

Supplementary Figure 8 The Neighbour-joining dendrogram generated based on simple matching dissimilarity coefficients using 867 SNPs for the 192 genotypes splitting the panel into two major subgroups A and M representing the Andean and Mesoamerican clades

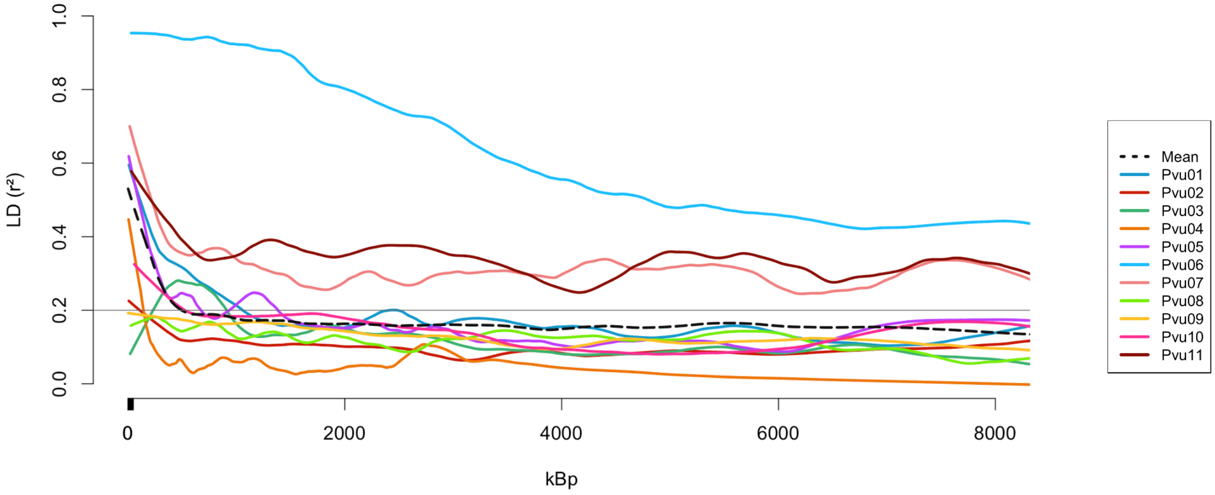

Supplementary Figure 9 Linkage disequilibrium decay by chromosome. LD plots for each chromosome and the mean LD for all 11 chromosomes

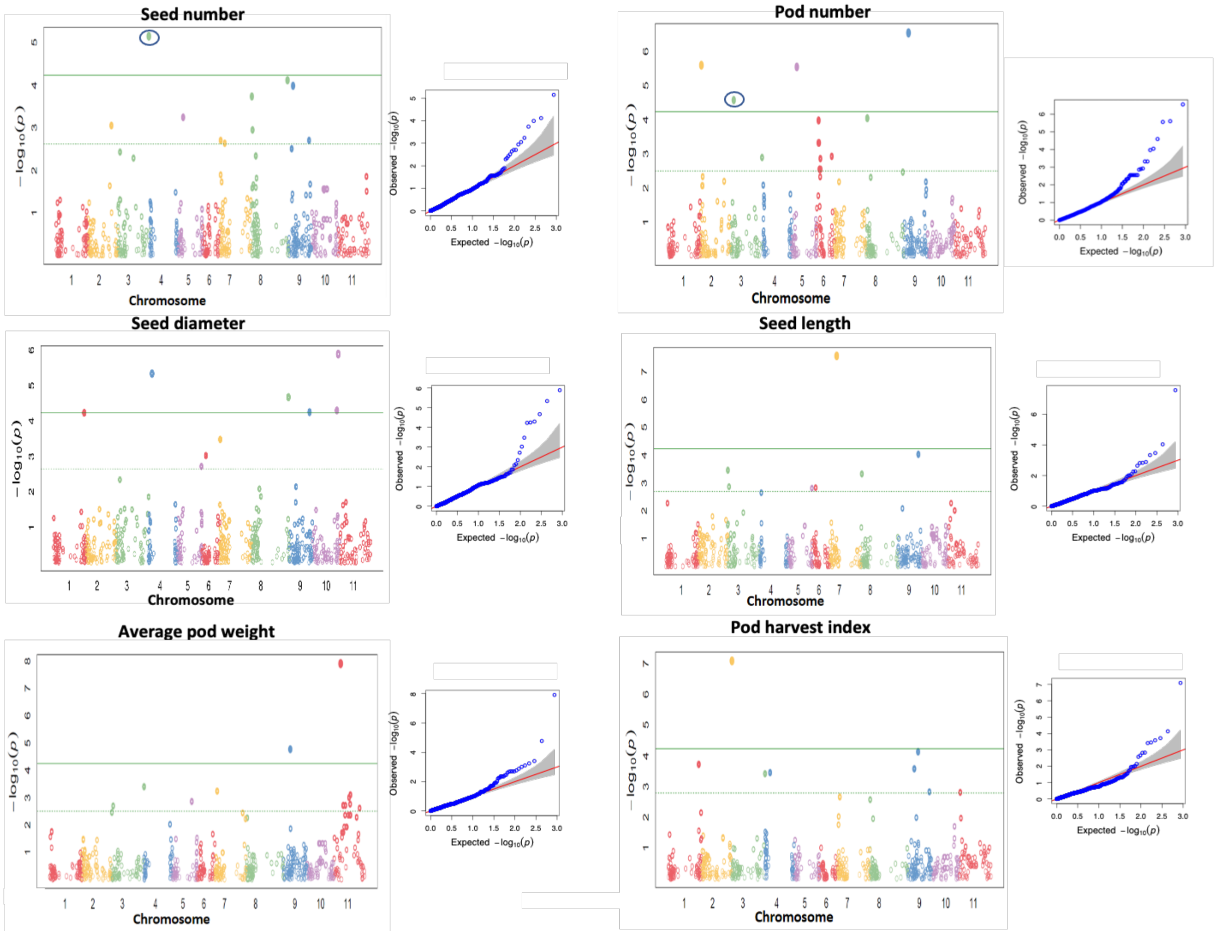

Supplementary Figure 10 Manhattan and QQ plots for the GWAS using FarmCPU for seed number, pod number, pod shattering, pod harvest index, average pod weight, seed diameter and seed length. The green line is the FDR cutoff value to call a significant peak. Significant peaks were observed on different chromosomes. Peaks enclosed in circles indicate SNPs associated with QTL overlapping with previously reported QTL

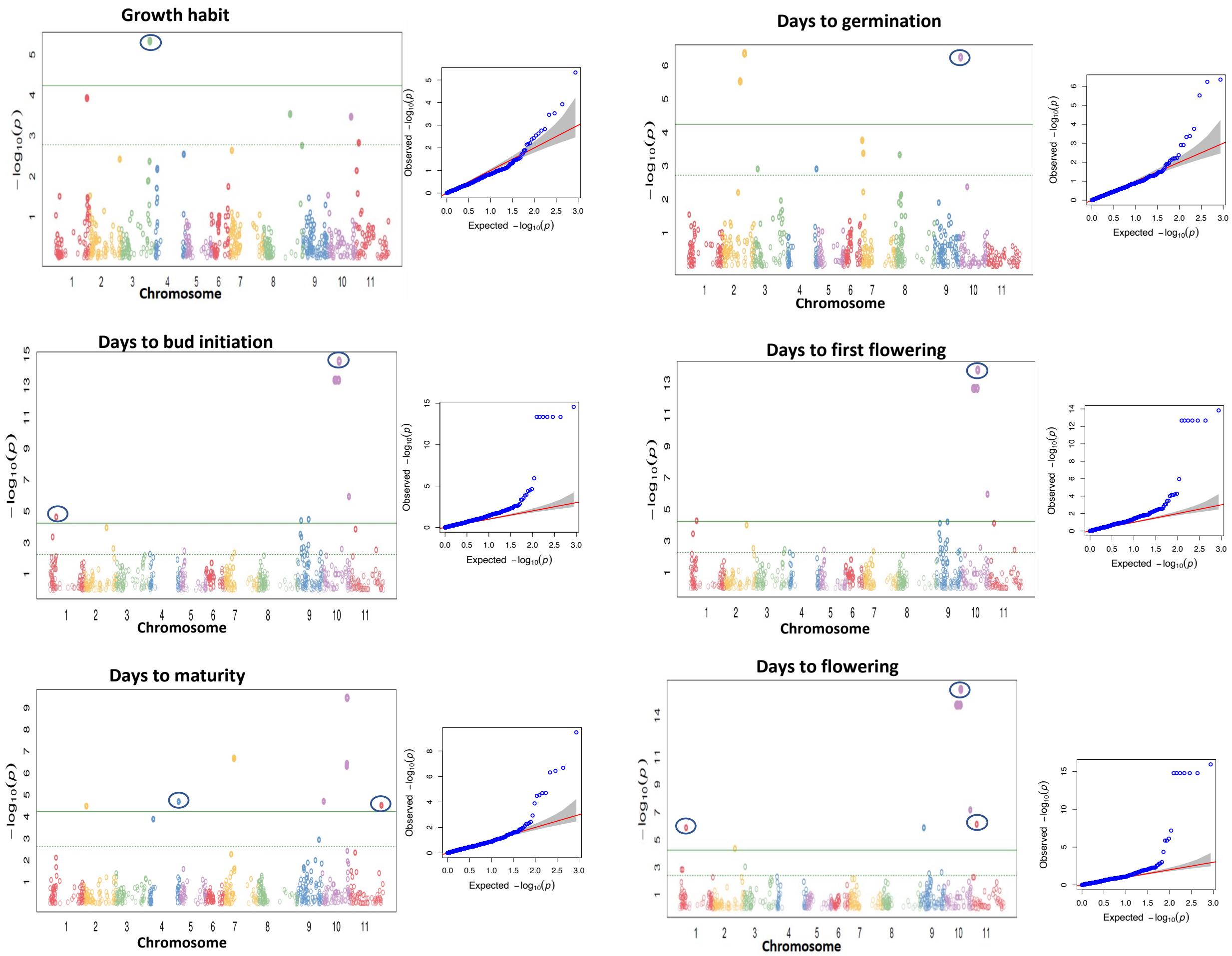

Supplementary Figure 11 Manhattan and QQ plots for the GWAS using FarmCPU for days to bud initiation, days to germination, days to first flowering, days to maturity, days to flowering and growth habit. The green line is the FDR cutoff value to call a significant peak. Significant peaks were observed on different chromosomes. Peaks enclosed in circles indicate SNPs associated with QTL overlapping with previously reported QTL

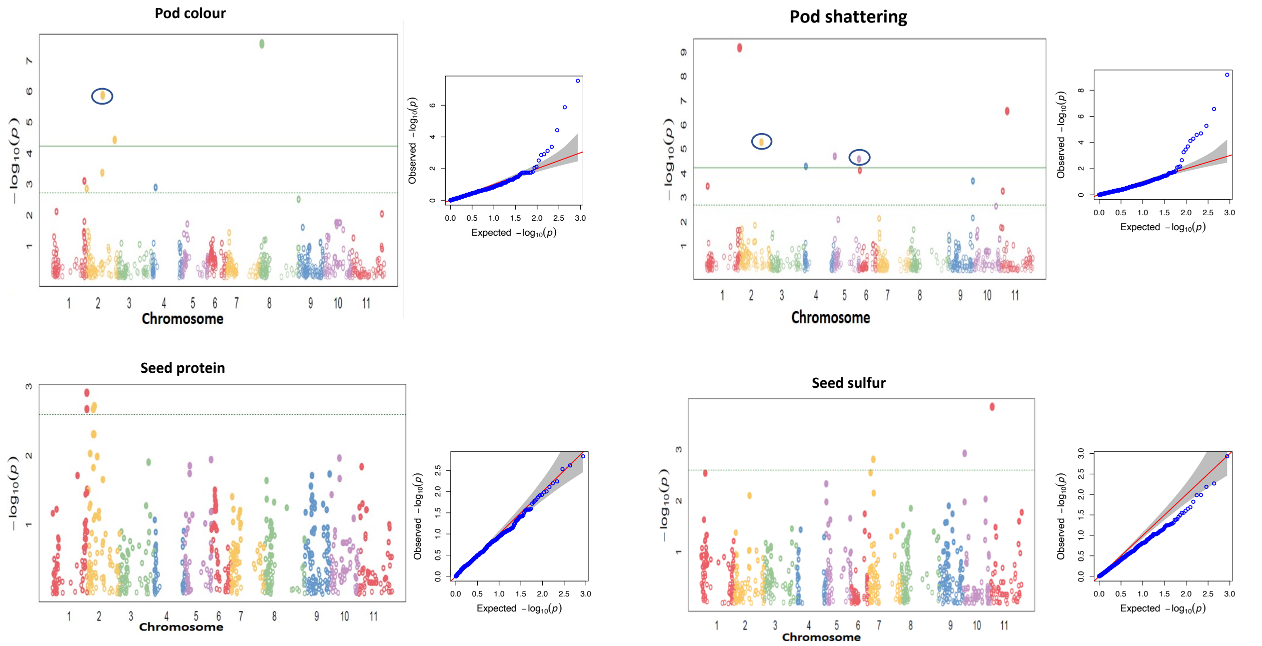

Supplementary Figure 12 Manhattan and QQ plots for the GWAS using FarmCPU for pod colour, pod shattering, seed protein and sulfur content. The green line is the FDR cutoff value to call a significant peak while the dotted line is an arbitrary threshold value to call a significant peak for seed quality traits. Significant peaks were observed on Pv01, Pv02, Pv07, Pv10 and Pv11. Peaks enclosed in circles indicate SNPs associated with QTL overlapping with previously reported QTL
